# O-GlcNAcylation of SPOP regulates colorectal cancer progression and ferroptosis by mediating β-catenin degradation

**DOI:** 10.1038/s41420-025-02832-y

**Published:** 2025-11-10

**Authors:** Xiuyuan Zhang, Yuwei Ding, Qizhen Ye, Saimeng Shi, Ning Zhu, Shanshan Weng, Jiaqi Chen, Wangxiong Hu, Ying Yuan

**Affiliations:** 1 Department of Medical Oncology, The Second Affiliated Hospital, Zhejiang University School of Medicine, Hangzhou, China; 2 Cancer Institute, Key Laboratory of Cancer Prevention and Intervention, Ministry of Education, The Second Affiliated Hospital, Zhejiang University School of Medicine, Hangzhou, China; 3 Research Center for Air Pollution and Health, School of Medicine, Zhejiang University, Hangzhou, China; 4 Zhejiang Provincial Clinical Research Center for CANCER, Hangzhou, China; 5 Cancer Center of Zhejiang University, Hangzhou, China; 6 Center for Medical Research and Innovation in Digestive System Tumors, Ministry of Education, Hangzhou, 310009 China

**Keywords:** Cancer therapeutic resistance, Cancer genetics

## Abstract

Current therapeutic approaches for colorectal cancer (CRC) face challenges such as recurrence and drug resistance. Ferroptosis, a novel form of cell death, is a promising therapeutic approach for CRC. SPOP plays an important biological role as a substrate-binding protein of the E3 ubiquitin ligase complex CRL3, but its therapeutic effects in CRC patients and its ability to modulate ferroptosis remain largely unknown. This study demonstrated that SPOP functions as a tumor suppressor in CRC and that SPOP inhibits the proliferation and metastasis of CRC cells and increases their sensitivity to ferroptosis. Transcriptome analysis suggested that Wnt signaling may be a potential target for the function of SPOP. Further data revealed that SPOP knockdown increased β-catenin protein levels, and the clinical data indicated that SPOP expression had the opposite effect on β-catenin protein levels. Molecular biology experiments suggest that SPOP promotes polyubiquitination and degradation of the K508 site of β-catenin. Interestingly, O-GlcNAcylation of SPOP reduces its protein stability and affects SPOP binding to β-catenin, and SPOP also promotes CRC ferroptosis by inhibiting the β-catenin/SLC7A11 axis. Combined treatment with the SPOP-targeted drug maprotiline and a ferroptosis inducer has synergistic antitumor efficacy in CRC cells and xenografts. Our study reveals the multifaceted function of SPOP in CRC, and the activation of SPOP may be a feasible strategy to increase the sensitivity of CRC to ferroptosis inducers.

## Introduction

Colorectal cancer (CRC), the most common malignant tumor of the digestive system, is one of the leading causes of cancer-related death [1]. Most CRC patients are diagnosed with metastasis and are in advanced stages [2], often require treatment, such as chemotherapy and immunotherapy, are resistant to multiple therapies, and have low survival rates [3]. An increase in the Wnt signaling pathway is observed in almost all CRC patients [4]; therefore, the Wnt signaling pathway may be a key regulatory mechanism driving CRC development.

Cullin-RING ligases (CRLs) are the most widely expressed E3 ubiquitin ligases in humans [5]. CRLs are composed of several parts, including a cullin scaffold protein, a RING-box protein (RBX1/2), an adaptor, and a substrate receptor. CRL3, which is implicated in cancer development, can specifically use the BTB structural domain as a substrate-regulated protein [6]. However, owing to the complex structure of CRL3, less is known about its mechanism of action. Speckle-type POZ protein (SPOP) is a substrate-binding receptor in CRL3, and SPOP can mediate both degradable and nondegradable polyubiquitination reactions of a variety of different biological substrates. The key to the ubiquitination function of SPOP is that SPOP has a MATH domain that recruits substrates by recognizing the SPOP-binding consensus (SBC) motif, as well as a BTB domain that is responsible for binding to CUL3 [7, 8]. SPOP has been reported to function as a tumor suppressor in most tumors [9–14], and recent studies have shown that SPOP can mediate STAT3 degradation, thereby inhibiting bladder cancer progression [15]. However, SPOP has a high mutation frequency in human tumor samples, with mutations occurring mainly in the MATH structural domain; these mutations cause inactivation of SPOP function, which leads to tumor progression, and SPOP mutations are more prevalent in prostate cancer and endometrial cancer [16]. There are few reports on whether SPOP regulates CRC development, whether these effects are dependent on ubiquitination, and what their specific substrates and pathways are; these areas require further exploration.

Ferroptosis is an iron-dependent form of programmed cell death caused by the loss of cellular redox homeostasis [17, 18], which can inhibit tumor cell proliferation, invasion, and metastasis, as well as increase the efficacy of radiotherapy and immunotherapy [18, 19]; thus, the discovery of ferroptosis highlights its potential as a therapeutic target for cancer treatment, providing elimination of malignant cancer cells as an alternative mechanism. To date, multiple tumor cell types have been shown to be susceptible to ferroptosis. SLC7A11, a subunit of Xc-, is responsible for transporting extracellular cysteine [20], and the ferroptosis inducer erastin reduces cysteine translocation into cells and induces ferroptosis by inhibiting SLC7A11 [21]. SLC7A11 is overexpressed in various types of cancers, including CRC [22]. Targeting SLC7A11 can trigger ferroptosis through lipid peroxidation and Fe^2+^ accumulation and restore sensitivity to chemotherapy [23].

In this study, we identified CRC-associated CRL3-SPOP, which plays a tumor suppressor role in CRC and can increase the susceptibility of CRC to ferroptosis. Mechanistically, SPOP binds to β-catenin and promotes its degradation and ubiquitination, which inhibits β-catenin binding to the promoter region of SLC7A11. In addition, SPOP can interact with the O-GlcNAc transferase OGT, which mediates the O-GlcNAcylation of SPOP, thereby decreasing the protein stability of SPOP and attenuating the binding of SPOP to β-catenin, which promotes CRC progression. Finally, we verified that maprotiline, a drug that targets SPOP, and a ferroptosis inducer play synergistic roles in preventing CRC growth, which provides a new direction for the future treatment of CRC.

## Results

### SPOP is expressed at low levels in CRC and is associated with a longer prognosis

**Fig. 1:**
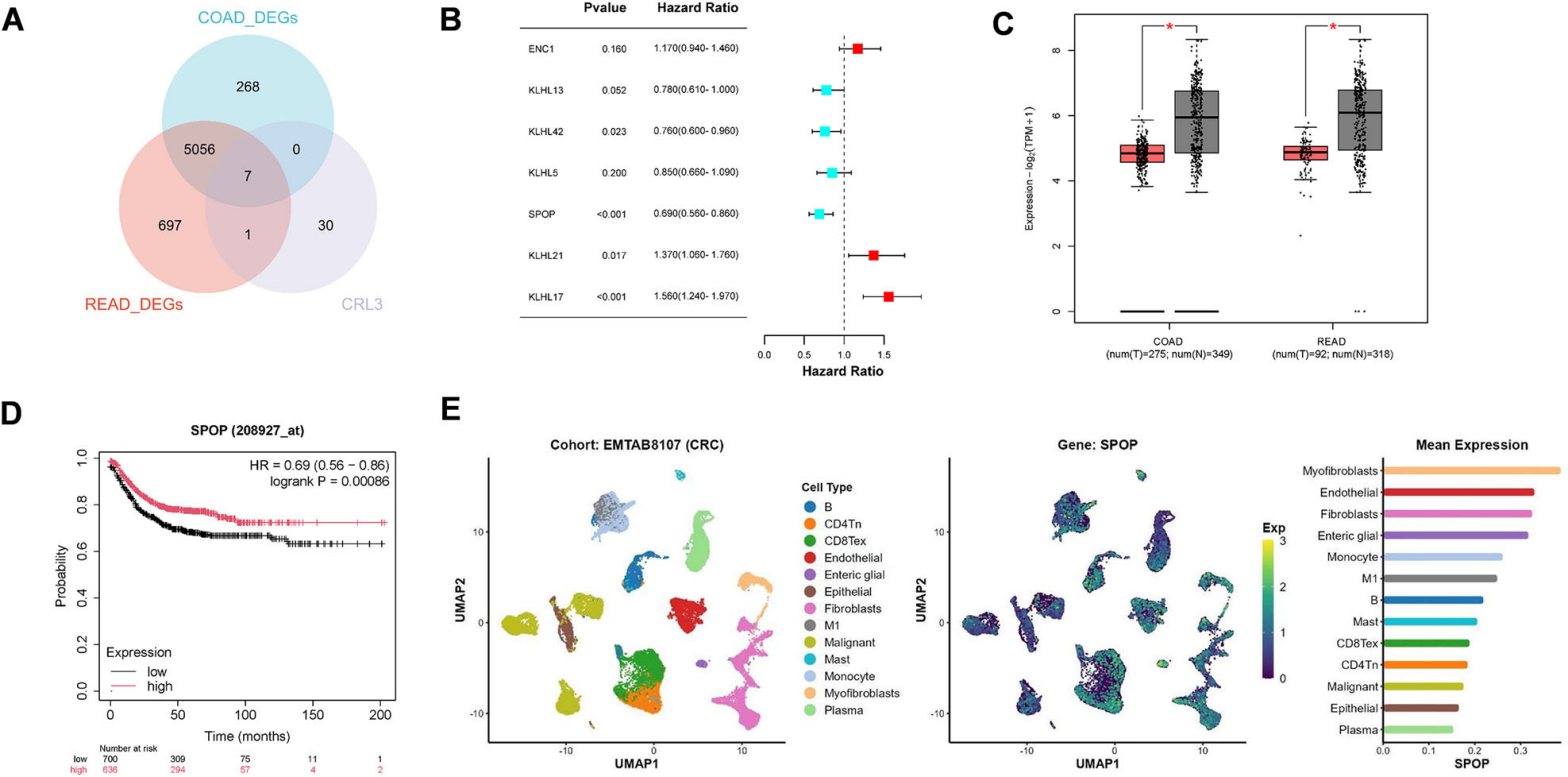
Low expression of SPOP is observed in CRC and is associated with a longer prognosis. **A** The Venn diagram displays the intersection of differentially expressed genes (DEGs) from the TCGA-COAD and TCGA-READ datasets with genes associated with CRL3. **B** Forest plot of candidate genes identified via univariate Cox regression analysis. **C** SPOP expression profiles of COAD and READ tumor samples and normal tissues based on the GEPIA. **D** Kaplan‒Meier analysis of the RFS of CRC patients stratified by SPOP expression was performed via the online tool Kaplan‒Meier Plotter. **E** t-SNE showing SPOP expression in different subpopulations of human CRC samples by single-cell sequencing.

We first collected a list of CRL3-related genes (*n* = 37) from the IUUCD database and then obtained differentially expressed genes (DEGs) through TCGA-COAD and TCGA-READ, which intersected to obtain seven candidate genes (Fig. 1A). Combined with the prognosis of these seven genes in CRC, we screened out the candidate gene SPOP (Fig. 1B). Using an online database, we found that SPOP was expressed at lower levels in CRC tissues than in normal tissues (Fig. 1C) and was associated with longer survival in CRC patients (Fig. 1D). To further explore the expression of SPOP in CRC subpopulations, we analyzed a CRC single-cell dataset (EMTAB8107), which revealed that the expression of SPOP was mainly concentrated in myofibroblasts and was relatively low in tumor cell subpopulations (Fig. 1E). These findings suggest that SPOP expression is downregulated in CRC and is correlated with a longer prognosis.

### SPOP inhibits CRC progression

**Fig. 2:**
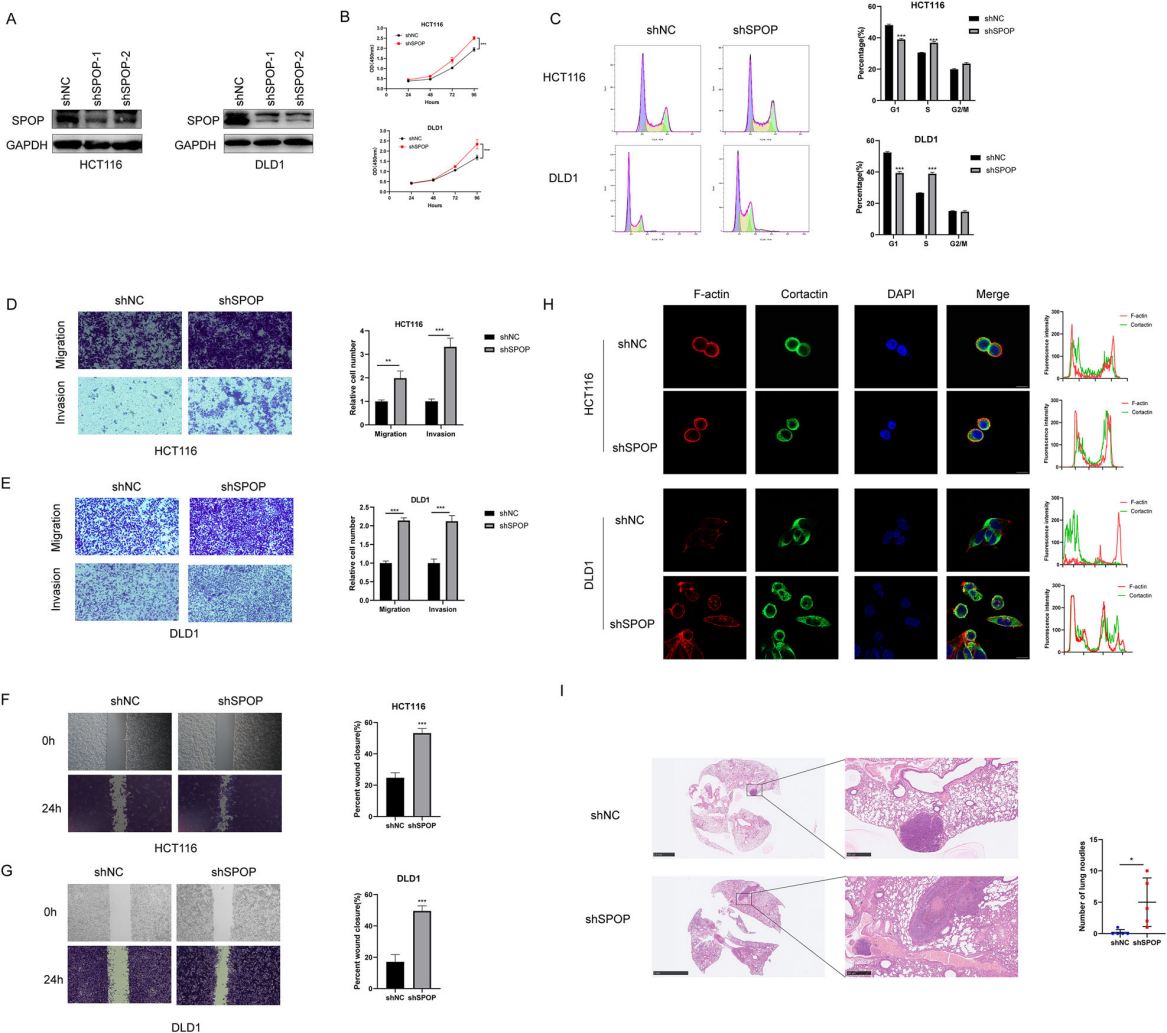
SPOP knockdown promotes CRC progression. **A** Lentiviral vectors expressing SPOP-specific shRNA were used to infect HCT116 and DLD1 cells, and WB analysis was performed to verify the knockdown efficiency. **B** Cell proliferation in these cell lines was measured using the CCK-8 assay. **C** Cell cycle phases were analyzed by flow cytometry, with representative images and quantitative results displayed. **D, E** Transwell assay. **F**, **G** Wound healing assay **H** Invadopodia were visualized by colocalization of cortactin and F-actin. **I** Representative images of lung sections from HCT116 cells and quantification of metastatic lung nodules; *N* = 5,scale bars, 500 µm.

To verify the biological function of SPOP in CRC, we first constructed SPOP-knockdown cell lines (Fig. 2A) and found that SPOP knockdown increased the proliferative ability of CRC cells via a CCK8 assay (Fig. 2B), while a cycle assay suggested that SPOP could change the cycle distribution of CRC cells (Fig. 2C). We also found that the knockdown of SPOP promoted the migration and invasion of CRC cell lines via wound healing and transwell assays (Fig. 2D–G). Invadopodia formation is characteristic of tumor metastasis, which generally manifests as colocalization of F-actin and Cortactin, and IF revealed that after SPOP was knocked down, the colocalization of Cortactin and F-actin was significantly increased (Fig. 2H), suggesting an increase in invadopodia. We then constructed a lung metastasis colonization model to verify the effect of SPOP on metastasis, and the results showed more metastatic foci in the shSPOP group (Fig. 2I), suggesting that SPOP could suppress metastasis. These results suggest that SPOP exerts tumorsuppressive effects in CRC.

### SPOP binds to β-catenin

**Fig. 3:**
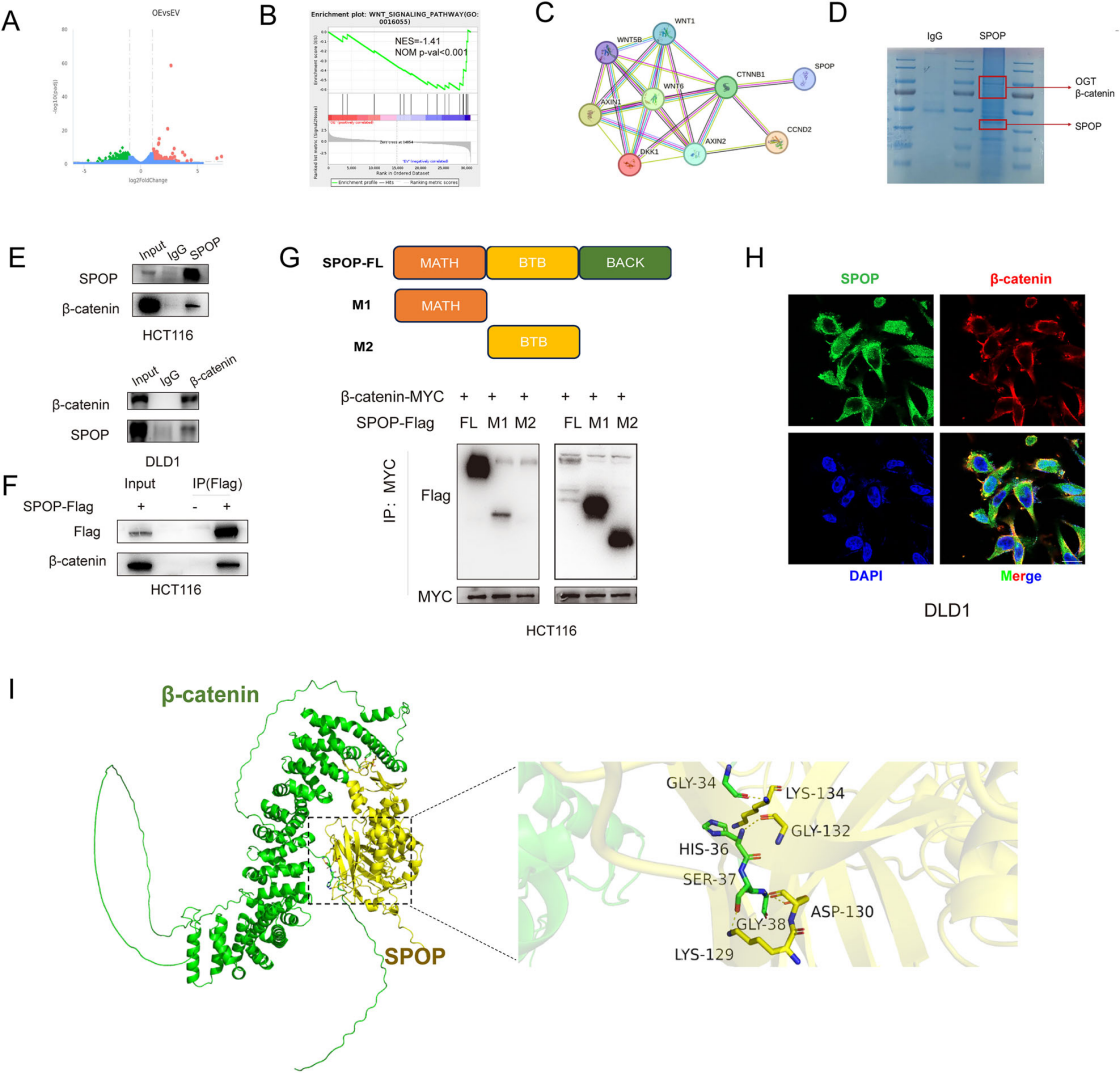
SPOP binds to β-catenin. **A** SPOP-overexpressing HCT116 cells subjected to transcriptome RNA-seq. **B** GSEA analysis based on RNA-seq data of the SPOP-overexpressing HCT116 cell line. **C** STRING database showing the PPI network of SPOP with Wnt pathway-related molecules. **D** Coomassie blue staining using anti-SPOP immunoprecipitation in HCT116 cells. The purified SPOP protein complexes were analyzed by MS. **E** Co-IP detection of the interaction between endogenous SPOP and β-catenin. **F** Co-IP analysis was performed to determine the interaction between exogenous SPOP and β-catenin. **G** Schematic representation of full-length and truncated SPOP mutants and the interaction of β-catenin with different SPOP mutants revealed by Co-IP in HCT116 cells. **H** IF assay for colocalization of SPOP and β-catenin in DLD-1 cells; scale bars, 20 µm. **I** Molecular docking model of the SPOP-β-catenin interaction.

To explore the mechanisms of SPOP action in CRC, we performed RNA-seq on SPOP-overexpressing HCT116 cells (Fig. 3A), The RNA-seq-based GSEA results suggested that the SPOP-overexpressing group had a reduced Wnt signaling pathway activity (Fig. 3B). Next, we analyzed the PPIs of Wnt signaling pathway-related molecules by the STRING database and found that SPOP was bound to β-catenin (encoded by CTNNB1) (Fig. 3C). In addition, we identified SPOP-bound proteins, which contain β-catenin (Fig. 3D), via IP‒MS; the binding peptides are shown in Fig. S1A.

We confirmed the existence of endogenous and exogenous interactions between SPOP and β-catenin via co-IP (Fig. 3E, F). The role of SPOP as an E3 ligase is dependent on the MATH structural domain, as it helps to recognize and bind various substrates.

To verify whether SPOP recognition of β-catenin is dependent on the MATH structural domain, we constructed MATH domain (M1)- and BTB domain (M2)-truncated mutants. The Co-IP results revealed that the truncation mutants with the MATH domain could bind to β-catenin, whereas the truncation mutants with the BTB domain could not bind to β-catenin (Fig. 3G), suggesting that SPOP recognition of β-catenin is dependent on the MATH structural domain.

Immunofluorescence further confirmed the colocalization of SPOP with β-catenin in CRC cells (Fig. 3H and Fig. S1B). In addition, molecular docking models predicted specific residues in the structural domain of SPOP that bind to β-catenin (Fig. 3I). These results suggest that the Lys129, Asp130, Gly132 and Lys134 residues of SPOP are key residues for binding to β-catenin. The G132V mutation of SPOP was reported to be a pathogenic missense mutation that increases the expression level of BRD4, a known substrate of SPOP [24]. Therefore, we constructed a SPOP-G132V point mutation plasmid, and the Co-IP results indicated that the SPOP-G132V mutation decreased binding to β-catenin (Fig. S2A), suggesting that the G132 site is a binding site for SPOP and β-catenin.

### SPOP regulates β-catenin expression

**Fig. 4:**
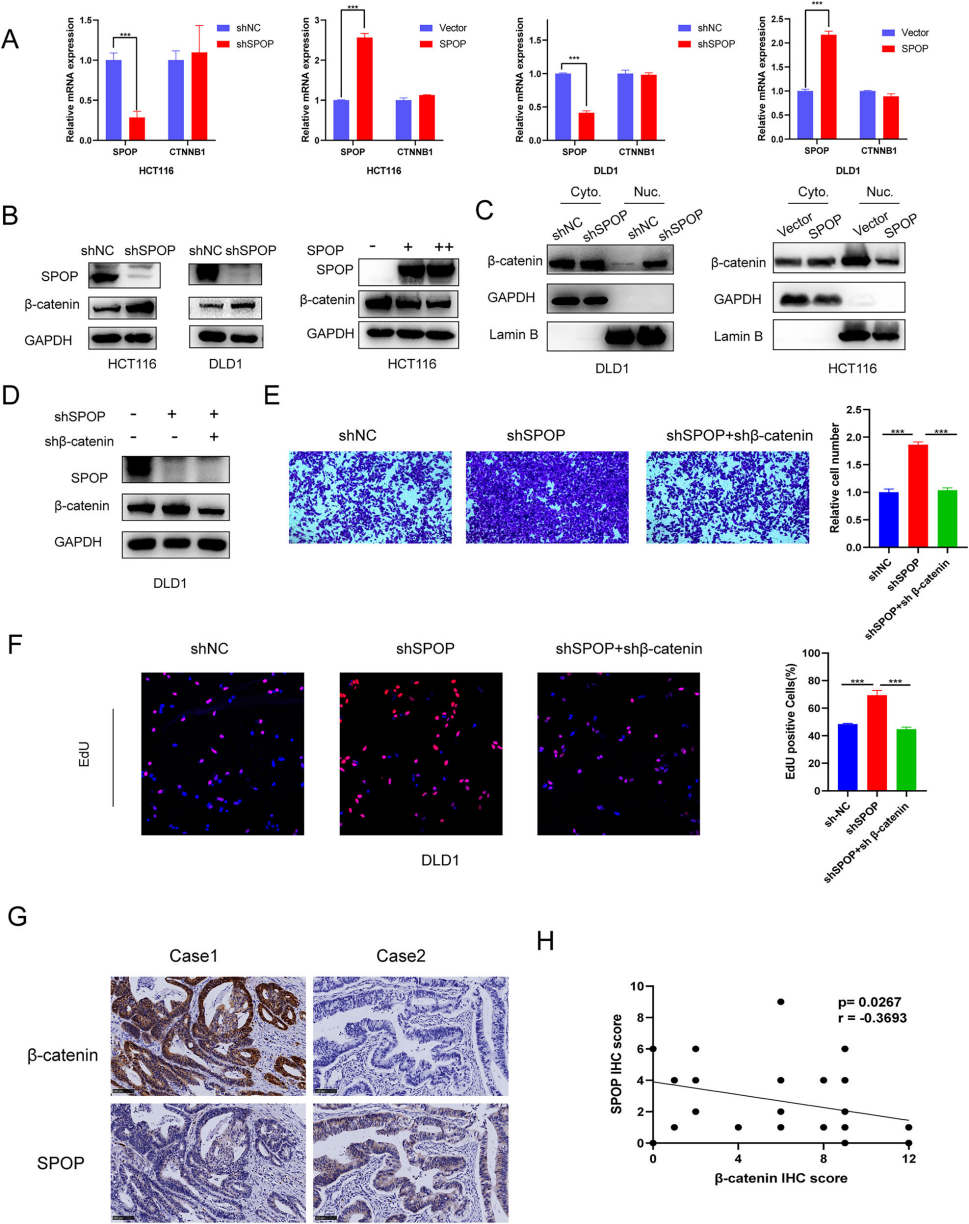
SPOP modulates β-catenin protein abundance without affecting its mRNA expression. **A** The mRNA levels of β-catenin in HCT116 and DLD1 cells transfected with shSPOP and those transfected with the SPOP overexpression plasmid were detected via qRT‒PCR. **B** WB for β-catenin expression in HCT116 and DLD1 cells transfected with shSPOP. **C** β-catenin expression in the cytoplasm and nucleus of shSPOP-transfected DLD-1 and SPOP-overexpressing plasmid-infected HCT116 cells was analyzed by WB. **D** DLD1 cells were co-transfected with shRNAs targeting SPOP and β-catenin, and subsequent protein expression changes were evaluated by WB. **E** Transwell assay of DLD1 cells. **F** EdU assay of DLD1 cells. **G** Representative IHC staining of SPOP and β-catenin in 36 CRC tumor tissues; scale bars, 100 µm. **H** Correlation analysis of SPOP and β-catenin expression in CRC tissues on the basis of the H score (Spearman correlation).

In CRC cells, neither overexpression nor knockdown of SPOP affected the mRNA levels of β-catenin (Fig. 4A). However, knockdown of SPOP increased of β-catenin protein levels, whereas the overexpression of SPOP decreased β-catenin protein levels (Fig. 4B), suggesting that SPOP may regulate β-catenin through a posttranslational modification (PTM) pathway.

Nuclear β-catenin can bind to TCF4 and costimulate the transcription of target genes to promote tumor progression [25, 26]. Therefore, we confirmed that nuclear β-catenin was increased in cells after SPOP was knocked down and decreased in cells after SPOP was overexpressed (Fig. 4C).

To verify the joint role of SPOP and β-catenin in CRC, we constructed cotransfected cell lines (Fig. 4D). The combined knockdown of SPOP and β-catenin attenuated the proinvasive and proliferative effects of SPOP knockdown on CRC cells, as shown by transwell and EdU assays (Fig. 4E, F).

IHC analysis was performed on CRC tissues to evaluate the protein expression levels of SPOP and β-catenin. The results demonstrated a pronounced negative association between the abundance of SPOP and β-catenin in tissues from CRC patients (Fig. 4G, H).

### SPOP promotes the ubiquitination of β-catenin

**Fig. 5:**
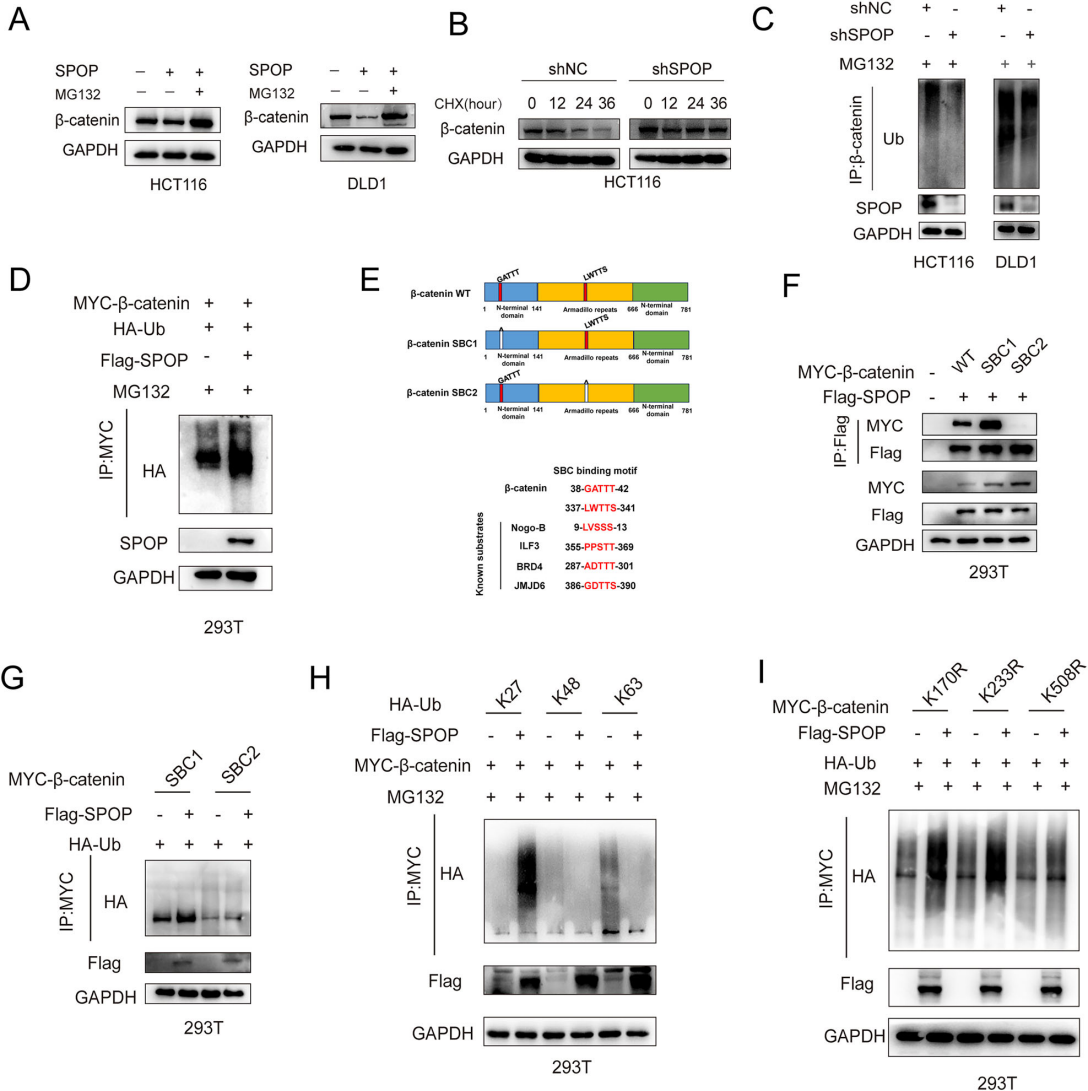
SPOP promotes β-catenin degradation and ubiquitination. **A** The expression of β-catenin in SPOP-overexpressing HCT116 and DLD1 cells was analyzed by WB after treatment with MG132 (10 μM, 6 h). **B** HCT116 cells transfected with shSPOP were treated with CHX, and samples were harvested at multiple time points for WB. **C** Total ubiquitination levels of β-catenin in SPOP-knockdown HCT116 and DLD1 cells transfected with the corresponding plasmids (48 h) and treated with MG132 (10 μM, 6 h). **D** 293 T cells were transfected with MYC-β-catenin, HA-Ub and Flag-SPOP. The cell lysates were subjected to IP analysis. **E** Sequence comparison of β-catenin with the SBC motif in known SPOP substrates. **F** 293 T cells were transfected with MYC-β-catenin-SBC1, MYC-β-catenin-SBC2 and Flag-SPOP. The cell lysates were analyzed via IP. **G** Ubiquitination assays via IP in 293 T cells transfected with MYC-tagged β-catenin SBC mutants, HA-Ub, and Flag-SPOP. **H** Analysis of β-catenin ubiquitination in 293 T cells co-expressing MYC-β-catenin, various HA-Ub linkage mutants (K27, K48, K63), and Flag-SPOP using IP. **I** IP analysis of ubiquitination in 293 T cells transfected with K-to-R β-catenin mutants (K170R, K233R, K508R), HA-Ub, and Flag-SPOP.

Considering that SPOP acts as an E3 ubiquitin ligase, and based on earlier experimental outcomes, we proposed that SPOP might downregulate β-catenin protein levels via the ubiquitin–proteasome system. To examine this possibility, we treated HCT116 and DLD1 cells overexpressing SPOP with the proteasome inhibitor MG132. The results revealed that MG132 could counteract the β-catenin degradation triggered by elevated SPOP expression (Fig. 5A). Additionally, using cycloheximide (CHX) to inhibit protein synthesis, we observed that SPOP knockdown in HCT116 cells led to a prolonged β-catenin half-life (Fig. 5B), indicating a role in protein turnover. Ubiquitination assays further supported this regulatory effect: silencing SPOP resulted in diminished β-catenin ubiquitination (Fig. 5C), whereas SPOP overexpression increased its ubiquitination level (Fig. 5D). Importantly, the SPOP G132V mutant failed to enhance β-catenin ubiquitination, suggesting a functional impairment associated with this mutation (Fig. S2B). These collective findings indicate that SPOP contributes to β-catenin ubiquitination and promotes its proteasomal degradation in CRC cells.Since the MATH domain of SPOP binds to β-catenin and the MATH domain recognizes the SBC motif of the substrate proteins [27], we inferred the two SBC motifs of β-catenin and constructed two β-catenin truncation plasmids (SBC1 and SBC2) (Fig. 5E). Co-IP revealed that the β-catenin-SBC2 mutant lost its ability to bind to SPOP (Fig. 5F) and that the ubiquitination level of the β-catenin-SBC2 mutant was significantly reduced (Fig. 5G).

Guo et al. reported that β-catenin can undergo K27-, K48-, and K63-linked polyubiquitination [28], so we performed ubiquitination experiments, and the results suggested that the overexpression of SPOP significantly increased the K27-linked polyubiquitination of β-catenin but did not affect the polyubiquitination of β-catenin linked to K48 and K63 (Fig. 5H). To identify the lysine sites on β-catenin recognized by SPOP, we predicted the lysine sites of β-catenin through the PhosphoSitePlus® database, selected the top 3 residues (K170, K233 and K508), and constructed mutants targeting these lysine residues to test our hypothesis. We found that only the K508R mutation blocked SPOP-mediated β-catenin ubiquitination (Fig. 5I). Taken together, SPOP promotes the ubiquitination of β-catenin at the K508 residue.

### O-GlcNAcylation of SPOP can affect the binding of SPOP and β-catenin

**Fig. 6:**
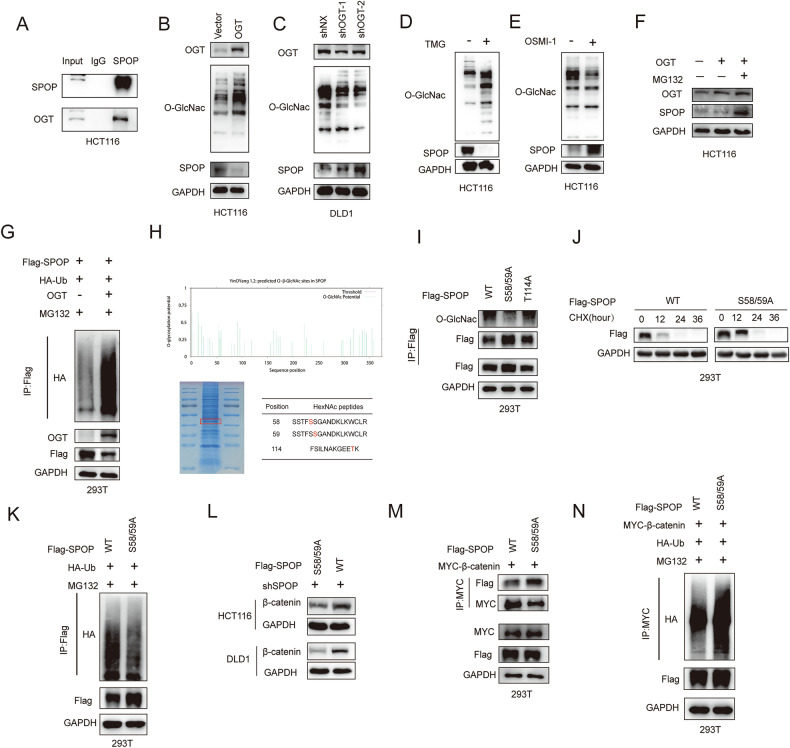
Binding of SPOP and β-catenin is affected by O-GlcNAcylation of SPOP. **A** Co-IP detection of the binding between endogenous SPOP and OGT. **B** SPOP protein levels were measured by WB in HCT116 cells after OGT overexpression. **C** WB was used to detect SPOP expression in DLD1 cells transfected with shOGT. **D** WB analysis was performed to assess SPOP expression in HCT116 cells treated with TMG. **E** SPOP protein levels in HCT116 cells following treatment with OSMI-1 were evaluated by WB. **F** The expression of SPOP in OGT-overexpressing HCT116 cells treated with MG132 (10 μm, 6 h) was analyzed by WB. **G** 293 T cells were transfected with HA-Ub, OGT overexpression plasmids and Flag-SPOP. The cell lysates were analyzed via IP. **H** The O-GlcNAc site on SPOP was identified via MS. **I** Lysates from 293 T cells transfected with Flag-SPOP-WT or its mutants (S58A/59 A and T114A) were subjected to IP analysis **J** 293 T cells transfected with Flag-SPOP or Flag-S58/59 A were treated with CHX, and all the cells were collected at different time points for WB analysis. **K** 293 T cells were transfected with Flag-SPOP-WT, Flag-SPOP-S58/S9A or HA-Ub. The cell lysates were analyzed via IP. **L** WB for β-catenin expression in CRC cells transfected with Flag-SPOP and Flag-SPOP-S58/59 A. **M** 293 T cells were transfected with Flag-SPOP-WT, Flag-SPOP-S58A/59 A and MYC-β-catenin, and the resulting cell lysates were analyzed via IP. **N** HA-Ub, MYC-β-catenin, and either Flag-SPOP or the S58/59 A mutant were introduced into 293 T cells via transfection. The cell lysates were analyzed via IP.

The IP-MS results suggested that OGT was a binding protein of SPOP, and since Zhou et al. reported that SPOP and OGT could bind to PDAC and 293 T cell lines [29], we verified this by Co-IP and found that SPOP and OGT could also bind to CRC cell lines (Fig. 6A). When OGT was overexpressed, the level of O-GlcNAc was increased, but the protein expression level of SPOP was decreased (Fig. 6B), and the opposite result was obtained after OGT was knocked down (Fig. 6C).

To further verify the regulation of SPOP expression by O-GlcNAc, the level of SPOP was upregulated after treatment with the OGT inhibitor OSMI-1 (Fig. 6D), whereas the level of O-GlcNAc was upregulated but the protein level of SPOP was downregulated in HCT116 cells after treatment with the OGA (the enzyme that removes O-GlcNAc) inhibitor TMG (Fig. 6E). These results suggest that SPOP protein levels in CRC are negatively regulated by O-GlcNAc levels.

Next, we sought to explore whether the ubiquitin‒proteasome pathway is involved in OGT-mediated SPOP degradation. HCT116 cells overexpressing OGT were treated with MG132. The results revealed that MG132 treatment blocked the degradation of SPOP caused by OGT overexpression (Fig. 6F), and the ubiquitination of SPOP was increased by OGT overexpression (Fig. 6G), suggesting that O-GlcNAc regulates SPOP degradation through a proteasome-dependent pathway.

Next, we used MS to identify O-GlcNAc sites on SPOP in HCT116 cells. We identified three possible glycosylation sites (S58, S59, and T114) (Fig. 6H). On the basis of these results, we generated different SPOP point mutants (S58/S9A and T114A) and examined their O-GlcNAc levels, and found that the S58/S9A mutant had significantly lower O-GlcNAc binding (Fig. 6I), suggesting that S58/S9A may act as an O-GlcNAc site on SPOP. To further confirm the stability of SPOP in relation to O-GlcNAc, we examined the half-life of the SPOP mutant S58/59 A, which presented a greater degradation rate than did SPOP-WT (Fig. 6J), and the ubiquitination level of the SPOP mutant S58/59 A was greater than that of SPOP-WT (Fig. 6K).

We demonstrated that SPOP binds to β-catenin and promotes its degradation, from which we speculated that the O-GlcNAcylation of SPOP could influence its relationship with β-catenin. We next found that the SPOP mutant S58/59 A had lower levels of β-catenin protein than SPOP-WT did and that the binding of the SPOP mutant S58/59 A to β-catenin was significantly greater than that of the SPOP-WT group was (Fig. 6L, M) and that β-catenin ubiquitination could be increased (Fig. 6N). Taken together, these results suggest that the O-GlcNAcylation of SPOP promotes β-catenin expression by inhibiting its binding to β-catenin and thus inhibiting β-catenin degradation.

### SPOP induces ferroptosis in CRC

**Fig. 7:**
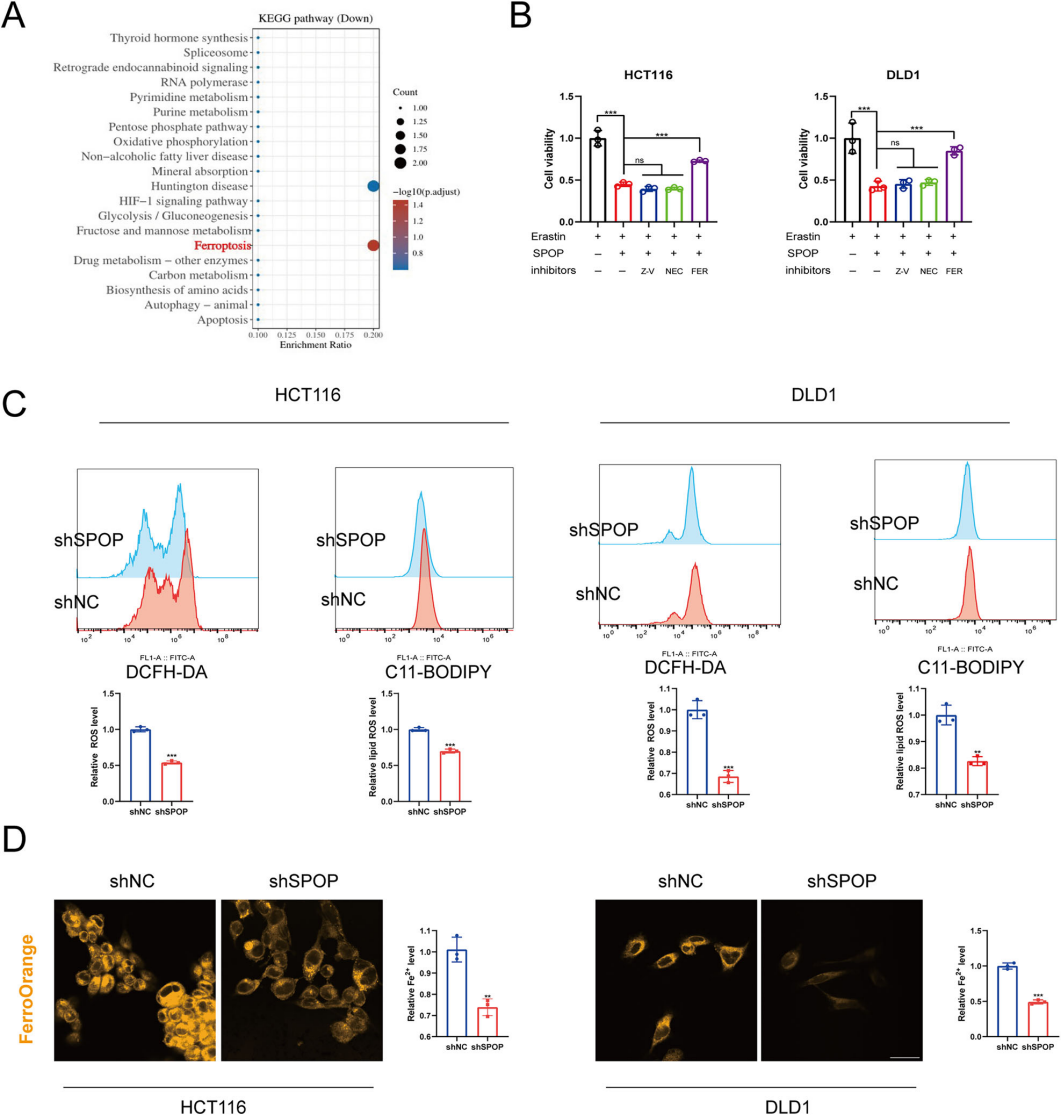
SPOP promotes ferroptosis in CRC. **A** Differential gene enrichment of the top 10 KEGG pathways. **B** CRC cells were transfected with the SPOP overexpression plasmid for 24 h. A total of 5000 transfected cells per well were inoculated into 96-well plates for 24 h and treated with 10 µm erastin alone or in combination with 2 µm Fer-1, 10 µm Z-VAD or 5 µm Nec for 48 h. **C** CRC cells transfected with shSPOP were treated with 10 µm erastin for 24 h, after which the cells were collected and stained with DCFH-DA and C11-BODIPY probes. ROS levels and lipid peroxidation responses were assessed via flow cytometry. **D** The intracellular Fe^2+^ concentration of CRC cells transfected with shSPOP was measured using FerroOrange; scale bars, 20 μm.

To further investigate the function of SPOP in regulating CRC growth, we performed KEGG analysis on the TCGA database, and the results revealed that ferroptosis was among the top 10 genes (Fig. 7A). Thus, we studied the association between SPOP and ferroptosis, and our results indicated SPOP overexpression significantly enhanced the erastin-induced inhibitory effects on the growth of CRC cells. This inhibitory effect was reversed by the ferroptosis inhibitor Fer-1, whereas it could not be reversed by other RCD (necroptosis and apoptosis) inhibitors, including Nec-1 and Z-VAD-FMK (Fig. 7B).

We examined the impact of SPOP on lipid ROS and ROS levels in CRC cells by C11-BODIPY and DCFH-DA and showed that the knockdown of SPOP resulted in significant decreases in lipid ROS and ROS levels in CRC cells (Fig. 7C). Fe2+ levels in CRC cells were detected with a FerroOrange probe, and the results indicated that after SPOP was knocked down, the Fe2+ levels in CRC cells significantly decreased (Fig. 7D). These results suggest that SPOP can induce sensitivity to ferroptosis in CRC cells.

### SPOP induces CRC ferroptosis by regulating the β-catenin/SLC7A11 axis

**Fig. 8:**
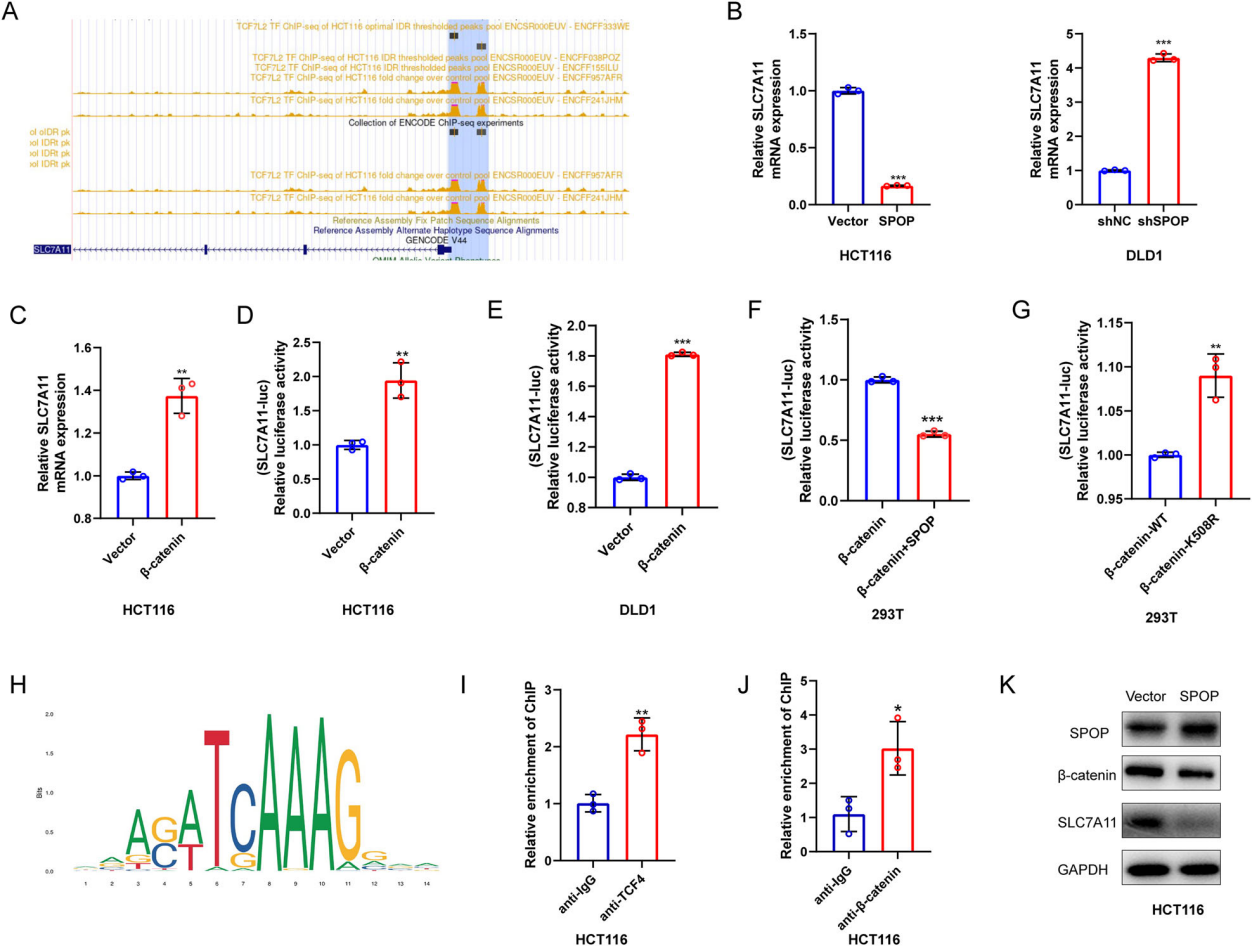
The SPOP/β-catenin/SCL7A11 axis regulates CRC ferroptosis. **A** ChIP-seq of TCF4 in the promoter region of SLC7A11. **B** qRT‒PCR was used to detect SLC7A11 mRNA levels in DLD1 cells transfected with shSPOP and SLC7A11 mRNA levels in HCT116 cells overexpressing SPOP. **C** The mRNA levels of SLC7A11 in HCT116 cells transfected with the β-catenin overexpression plasmid were detected via qRT‒PCR. **D–G** Dual luciferase reporter assay of SLC7A11 promoter activity. **H** TCF4-binding motif of SLC7A11 obtained from JASPAR. **I** ChIP‒qPCR analysis demonstrated the binding of TCF4 to the SLC7A11 promoter. **J** ChIP‒qPCR results also confirmed the association between β-catenin and the SLC7A11 promoter region. **K** HCT116 cells were transfected with the SPOP overexpression plasmid, and the cells were collected for WB analysis.

To elucidate the mechanism by which SPOP regulates ferroptosis in CRC cells, we first analyzed the ChIP-Atlas online database. Interestingly, the promoter sequence of SLC7A11 in the HCT116 cell line displayed a binding peak of β-catenin/TCF4 (Fig. 8A), and qPCR suggested that SPOP could downregulate the mRNA level of SLC7A11 (Fig. 8B), indicating that SPOP regulates SLC7A11 through the transcriptional pathway. Our previous study suggested that SPOP binds to β-catenin; thus, we speculated that SPOP regulates SLC7A11 expression through β-catenin (Fig. 8C). We first showed that the upregulation of β-catenin increased the mRNA level of SLC7A11. Through dual-luciferase reporter assays, we further confirmed that β-catenin enhances the transcriptional activity of the SLC7A11 promoter. In contrast, SPOP overexpression downregulated the β-catenin-upregulated SLC7A11 promoter activity of SLC7A11 (Fig. 8F), and the upregulation of SLC7A11 promoter activity by β-catenin K508R was more pronounced than that caused by β-catenin WT (Fig. 8G), suggesting that SPOP can modulate the effect of β-catenin on the promoter activity of SLC7A11. The JASPAR database predicted β-catenin/TCF4 binding to the SLC7A11 motif (Fig. 8H); thus, we performed ChIP‒qPCR to verify the binding of β-catenin/TCF4 to the promoter region of SLC7A11 (Fig. 8I, J), which was consistent with the database results. SPOP overexpression reduced the protein levels of β-catenin and SLC7A11 (Fig. 8K), suggesting that the SPOP/β-catenin/SLC7A11 axis may regulate ferroptosis.

### Maprotiline (MAP) and the ferroptosis inducer IKE have synergistic anticancer effects

MAP binds to SPOP and can target SPOP to regulate the ubiquitination of PD-L1, has synergistic anticancer effects with ICIs, and increases the expression level of SPOP. We found that MAP reduced the proliferation and migration ability of CRC cell lines (Fig. S3A–C) and increased the level of ubiquitinated β-catenin (Fig. S3D). Using flow assays, we found that MAP increased lipid ROS levels in CRC cells in a dose-dependent manner (Fig. S3E), suggesting that MAP has an anticancer effect on CRC.

**Fig. 9:**
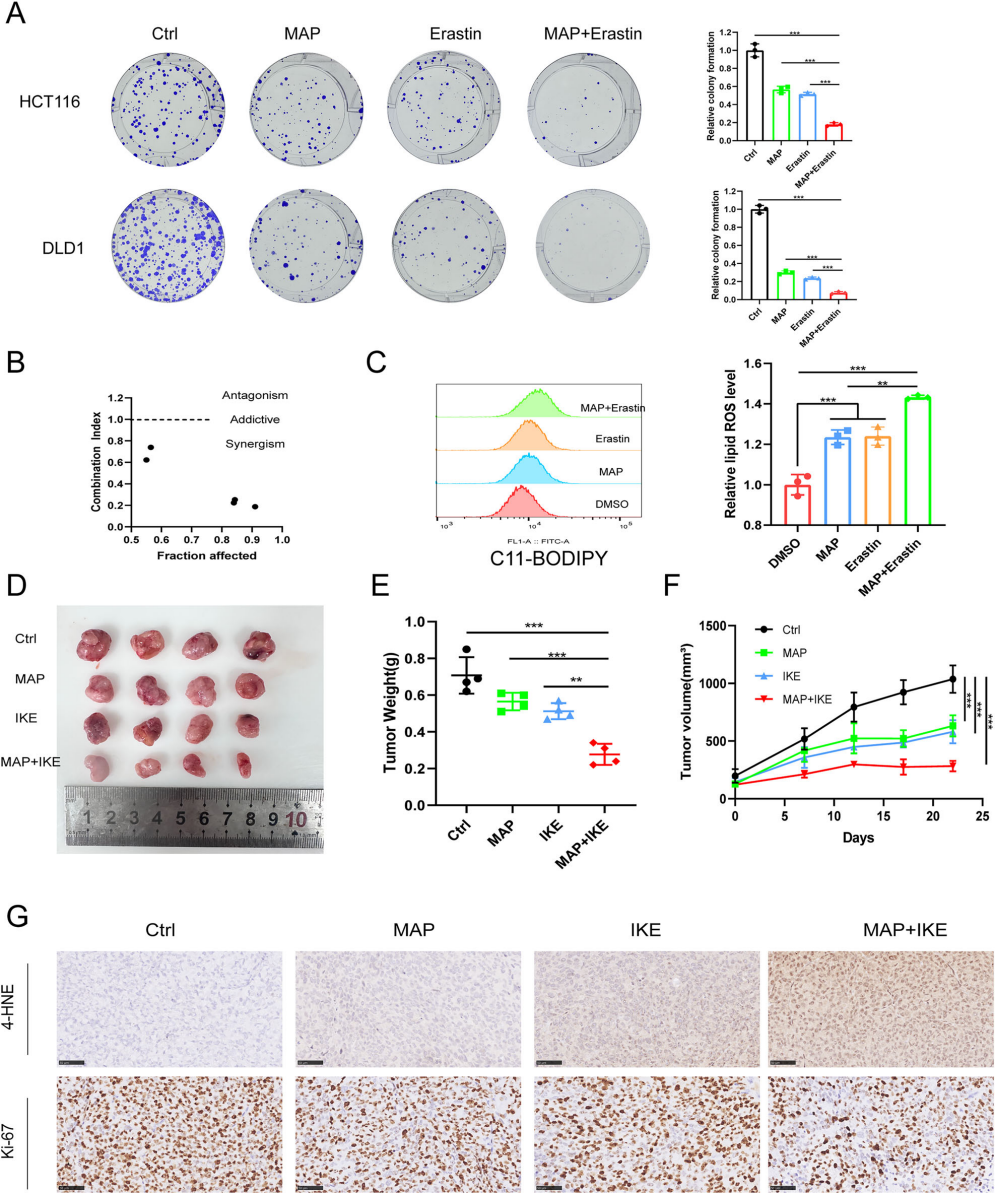
MAP and ferroptosis inducers have synergistic anticancer effects. **A** Colony formation assays were performed on CRC cells using different concentrations of MAP, ferroptosis incucer and their combinations. **B** The combination index of MAP and erastin in HCT116 cells was calculated via CalcuSyn software. **C** C11-BODIPY probe staining for assessing lipid ROS levels. **D** Representative images of subcutaneous tumors in each group (*n* = 4). **E** Tumor weight. **F** Tumor volume growth curves. **G** IHC staining of tumors in the indicated groups with anti-4-HNE and anti-Ki67 antibodies; scale bars, 50 μm.

Next, we explored whether MAP can increase the sensitivity of CRC to ferroptosis by combining MAP with a ferroptosis inducer. In vitro experiments revealed that the combination of MAP and erastin significantly inhibited the growth of CRC cells compared with the single agent alone (Fig. 9A), and the combination index of MAP and erastin was less than 1 (Fig. 9B), suggesting that the combination had a synergistic effect [30]. The flow cytometry results also revealed that, compared with the Erastin alone group, the combination group presented significantly increased lipid ROS levels (Fig. 9C).

We verified the effect of the combination by establishing a xenograft model, and after HCT116 injection for 1 week, we continued the treatment for 3 weeks by daily oral administration of MAP (30 mg/kg) and intraperitoneal injection of IKE (40 mg/kg) every 2 days. Compared with IKE alone, the combination of MAP with IKE significantly reduced the tumor volume and weight and slowed tumor growth (Fig. 9D–F).

The results of IHC staining suggested that the combination of MAP and IKE increased the expression level of 4-HNE (a marker of ferroptosis) and decreased the Ki67 ratio in tumor tissues, which was consistent with previous results (Fig. 9G). In conclusion, the combination of MAP and IKE enhances sensitivity to ferroptosis in vivo.

We further evaluated the biosafety of the combination of MAP and IKE in vivo, and the results revealed that there was no significant change in body weight between the treatment groups (Fig. S4A). Histopathological examination of major organs via H&E staining revealed that no significant morphological changes occurred in the groups (Fig. S4B). In conclusion, MAP and the combination of MAP and IKE demonstrated favorable biosafety profiles in vivo, supporting its potential for further therapeutic applications.

## Discussion

In this study, in terms of function, we found that SPOP plays a tumor suppressor role in CRC. SPOP was expressed at low levels in CRC samples, correlated with longer survival, and was negatively correlated with the protein expression level of β-catenin in CRC. SPOP inhibited the proliferative and metastatic ability of CRC cells and enhanced the sensitivity of CRC to ferroptosis. In terms of molecular mechanisms, SPOP can bind to β-catenin and promote ubiquitination and degradation of the β-catenin K508 site, thereby inhibiting downstream SLC7A11 activation. In addition, SPOP can undergo O-GlcNAcylation, which affects the protein activity and stability of SPOP and thus downstream pathways. In terms of clinical applications, we found that the SPOP-targeted drug maprotiline has a synergistic effect on inhibiting CRC growth in combination with the ferroptosis inducer IKE.

Ubiquitination modification is a common posttranslational modification (PTM) process of proteins in organisms, and abnormalities in the ubiquitin–proteasome system are also closely related to tumor formation and metastasis [16]; therefore, identifying the target proteins involved in the regulation of E3 ubiquitin ligases is important, and the discovery of proteolysis-targeting chimeras (PROTACs) is a major hotspot in current tumor research. Ferroptosis is a promising therapeutic tool for highly drug-resistant colorectal cancer, and this study explored a new regulatory mechanism of ferroptosis based on posttranslational modifications. New strategies for targeted therapy of CRC

We found that SPOP interacts with β-catenin in CRC and that β-catenin, a key player in the Wnt signaling [31], is not only intimately linked to carcinogenesis, tumor progression, and metastasis, but also plays a significant role in ferroptosis. We also discovered that SPOP interacts with β-catenin in CRC. In gastric cancer, The β-catenin/TCF4 transcriptional complex induces GPX4 expression, thereby inhibiting ferroptosis [32]. In CRC, MsrB/β-catenin activates GPX4 and ultimately inhibits ferroptosis [33]. In prostate cancer, SPOP can inhibit ferroptosis by promoting Jumonji domain-containing 6 (JMJD6) proteasomal degradation, thereby inhibiting the coordinated enhancer‒promoter loop interaction between JMJD6 and ATF4 and suppressing the expression of SLC7A11, a key gene for glutathione biosynthesis, thus promoting ferroptosis [34]. We found that SPOP specifically induced β-catenin to undergo polyubiquitylation, reduced its protein stability and thus inhibited the binding of β-catenin to the promoter sequence of SLC7A11, suppressed SLC7A11 expression, and promoted CRC ferroptosis. Our findings emphasize the broad function of SPOP as an E3 ubiquitin ligase in the regulation of target protein degradation and explore the mechanism by which SPOP regulates ferroptosis in CRC. Although previous studies have shown that SPOP promotes tumor progression by activating the β-catenin/TCF4 complex in clear cell renal cell carcinoma [35], we found that SPOP inhibits CRC progression by degrading β-catenin. This contradictory observation may be related to tumor heterogeneity. To our knowledge, this is the first study to demonstrate that SPOP binds to β-catenin, increases its ubiquitination level, and thereby causes its degradation, thus revealing a novel mechanism.

O-GlcNAcylation is a PTM of serine or threonine residues that occurs in intracellular proteins and is regulated by two enzymes: OGT and OGA [36]. O-GlcNAcylation has been reported to be correlated with cancer therapeutic resistance and to interact with ubiquitination to some extent [37]. The process of ubiquitination can alter protein stability and intracellular distribution, which may indirectly impact the glycosylation of ENO1 at serine 249. This modification could disrupt the interaction between ENO1 and PD-L1, thereby diminishing the association of PD-L1 with the E3 ubiquitin ligase STUB1. As a result, PD-L1 becomes more stable, ultimately facilitating the progression of CRC [38]. In this study, we found that SPOP can bind to OGT, which can regulate the ubiquitination level and protein stability of SPOP. By LC‒MS, we identified the O-GlcNAcylation modification sites S58 and S59 of SPOP in CRC, and the O-GlcNAcylation modification of SPOP can affect the binding of SPOP to β-catenin and change the ubiquitination level of β-catenin, these findings underscore the pivotal role of O-GlcNAcylation in modulating the functional activity of SPOP within the context of CRC.

Previous studies have shown that the combination of MAP and an anti-CTLA4 antibody significantly enhances antitumor efficacy in CRC patients and lung cancer patients because MAP binds to SPOP through the ARG70 site, increases the protein expression level of SPOP, and thus induces PD-L1 ubiquitination. In HCC, the combination of MAP and the ferroptosis inducer sorafenib significantly increased the inhibitory effect on HCC [39], suggesting that MAP has a sensitizing effect on ferroptosis. This study revealed that MAP modulates β-catenin ubiquitination, thereby inhibiting CRC cell proliferation and migration, and enhancing lipid peroxidation, collectively contributing to its antitumor activity. An in vivo study revealed that MAP, in combination with IKE, has a synergistic effect on CRC and has a favorable safety profile.

Although this study revealed that O-GlcNAcylation affects the protein stability of SPOP and regulates its ubiquitination level, the specific E3 ligases responsible for SPOP ubiquitination require further elucidation. In addition, although it was verified that MAP and IKE had a synergistic effect on the inhibition of CRC growth, this finding requires verification by the construction of more clinically meaningful organoid and PDX models.

## Materials and methods

### Cell culture

The human CRC cell lines (HCT116 and DLD1) and 293 T cells were obtained from the Stem Cell Bank, Chinese Academy of Sciences. Following receipt, the cells were cultured in DMEM (Gibco) supplemented with 10% fetal bovine serum (FBS; Gibco, 10099). The cells were incubated with 5% CO2 at 37 °C.

### Mass spectrometry (MS) Analysis

HCT116 cells transfected with Flag-SPOP plasmid were used to identify novel SPOP binding proteins. SPOP proteins were immunoprecipitated at 4 °C using anti-SPOP antibody and protein A/G beads (HY-K0202). LC-MS/MS analysis was performed using by Cosmos Wisdom Biotech Co., Ltd., Hangzhou, China. Mass spectra were processed and searched using Proteome Discoverer (version 2.4, Thermo Scientific) against the human Swissprot protein database (release 2023_09).

### Animal experiments

Animal experiments were approved by the Ethics Committee of the Second Affiliated Hospital of Zhejiang University School of Medicine (Approval No. 2024-120). Female BALB/c nude mice (4-5 weeks old) were purchased from Shanghai BK Laboratory Animal Co., Ltd. HCT116 cells were digested and mixed with Matrigel at a ratio of 1:1, and then 4 × 106/100 µL of cells were injected subcutaneously. When the tumour volume reached 100 mm3, the mice were divided into four groups and treated with the corresponding drugs. Tumor volumes were calculated using the following formula: tumor volume (mm3) = L × S × S/2.

### Statistical analysis

All statistical analyses were performed using GraphPad Prism 8 software (San Diego, CA, USA). Data are expressed as mean ± standard deviation (SD). Unpaired t-test was used to compare data between two groups. One-way ANOVA with Tukey’s test was used to compare data between multiple groups. Correlations were tested by Spearman’s correlation coefficient. *P* < 0.05 was considered statistically significant.

Details of the experiments are provided in the Supplementary Material.

## Supplementary information

Supplementary material

Supplementary Table S3

Original western blots

## Data Availability

The data that support the findings of this study are available from the corresponding author upon reasonable request.
