# Supplementary material for: O-GlcNAcylation of SPOP regulates colorectal cancer progression and ferroptosis by mediating β-catenin degradation
Source: Cell Death Discov. 2025 Nov 10;11:526. doi: 10.1038/s41420-025-02832-y (PMC12603323; doi:10.1038/s41420-025-02832-y)
Supplement: Supplementary file 1 — Supplementary material [file 41420_2025_2832_MOESM1_ESM.docx]

## Supplementary methods

### Antibodies and reagents

Antibodies against SPOP (#16750-1-AP, Proteintech, 1:2000), β-catenin (#ab32572, Abcam, 1:5000), OGT (#1576-2-AP, Proteintech, 1:1000), SLC7A11 (#26864-1-AP, Proteintech, 1:1000), TCF4/TCF7L2 (#13838-1-AP, Proteintech, 1:1000), O-GlcNac (#ab2739, Abcam, 1:1000), GAPDH (#60004–1-Ig, Proteintech, 1:20000), HA-tag (#ab18181,Abcam, 1:1000), MYC-tag (#16286-1-AP, Proteintech, 1:2000), and Flag-tag (#66008-4-Ig, Proteintech, 1:5000) were used. MG132 (#S2619), cycloheximide (#S7418), and erastin (#S7242) were purchased from Selleck (Shanghai, China). Maprotiline hydrochloride (MAP, HY-B0444), imidazole ketone erastin (IKE, HY-114481), necrostatin-1 (HY-15760), ferrostatin-1 (Fer-1, HY-100579), Z-VAD-FMK (HY-16658B), OSMI-1 (HY-119738) and thiamet G (TMG, HY-12588) were purchased from MedChemExpress (MCE, Shanghai, China).

### Transcriptome sequencing

RNA-seq was performed on SPOP-overexpressing HCT116 cells (three replicates per group). Total RNA was extracted via TRIzol reagent, and transcriptome sequencing was performed via NOVOGENE (Beijing, China). The RNA-seq data are provided in Supplementary Table S3.

### Plasmids and shRNA infection

Plasmids for the overexpression of Flag-SPOP, MYC-β-catenin, HA-Ub, and OGT and appropriate cDNA sequence fragments were obtained from You Bao Biotechnology (Changsha, China). For transfection, LipoD293 was used. For infection, shRNAs together with pMD2.G and psPAX2 were employed to produce lentiviral particles in 293T cells. The virus-containing cell culture medium was collected and used to infect CRC cells in the presence of 10 µg/mL polybrene. Puromycin (2 µg/mL) selection was performed to screen the successfully infected cells for 2 weeks. The sequences of shRNAs are listed in Supplementary Table S2.

### Quantitative Real-time PCR (qRT-PCR)

Total RNA from CRC cells was isolated using the RNA-Quick Purification Kit (ES Science, Shanghai, China). Reverse transcription was performed using Hifair III 1st Strand cDNA Synthesis Super Mix for qPCR kit (Yeasen, China), qRT-PCR was performed using the Hieff qPCR SYBR Green Master Mix kit(Yeasen, China). The primers used are listed in Supplementary Table S1.

### Immunofluorescence (IF) assay

HCT116 and DLD1 cells were seeded evenly on confocal culture dishes and incubated for appropriate time. Then they were fixed with 4% paraformaldehyde, permeabilized with 0.1% Triton X-100 and blocked with 5% BSA. The cells were then incubated with primary antibody at 4°C overnight. After staining the nuclear with DAPI, images were acquired under a confocal microscope (Carl Zeiss).

### Western blotting

Total protein was extracted from CRC cells and quantification of protein concentration using the Pierce BCA Protein Assay Kit (Thermo Fisher Scientific). Samples were assayed on SDS-PAGE gels, then proteins were transferred to PVDF membranes and blocked with 5 % nonfat milk for 1 hour, followed by incubation with primary antibody for overnight at 4 °C. Following incubation with a secondary antibody conjugated to HRP for 1 h at room temperature. For co-immunoprecipitation (IP), cells were lysed in IP lysis buffer containing 1% protease inhibitor cocktail for 30 min. The supernatant was collected and co-cultured with protein A/G magnetic beads and primary antibodies or IgG at 4 °C overnight. After five washes, the beads were mixed with 1X SDS-PAGE sample bufferr and boiled for 10 min. These samples were then subjected to Western blotting analysis.

### Co-immunoprecipitation (Co-IP) Assays and Ubiquitination Assays

For Co-IP, cells were lysed in IP lysis buffer containing 1% protease inhibitor cocktail for 30 min. The supernatant was collected and co-cultured with protein A/G magnetic beads and primary antibodies or IgG at 4 °C overnight. After five washes, the beads were mixed with 1X SDS-PAGE sample bufferr and boiled for 10 min. These samples were then subjected to Western blotting analysis.

For ubiquitination assays, cells transfected with the corresponding plasmids were treated with 10 µM MG132 for 6 hours before harvest. Cells were divided into two parts: input group and immunoprecipitation (IP) group. Each group was incubated with the corresponding label-specific antibody. Ubiquitinated protein levels were subsequently analyzed according to standard immunoprecipitation and Western blotting procedures

### Cell Viability Assay

Cell viability was analyzed using the cell counting KIT-8 (CCK-8) assay (Boster, China). For drug treatment, 5000 transfected cells were inoculated in 96-well plates for 24 hours and treated with different drugs for 48 hours. CCK-8 was added to each well, and absorbance was measured at 450 nm using a microplate reader after 2 h of incubation.

### Colony Formation Assay

Cells were plated in a 6-well plate at a density of 1000 cells per well, and cultured for approximately 14 days. Cells were rinsed twice with PBS, fixed with 4% paraformaldehyde for 15 min, and then stained with f 0.1% crystal violet solution for 30 min.

### Wound Healing Assay

Seed the treated cells into the Culture-Insert 2 well. After the cells adhere, remove the Culture-Insert, then incubate the cells in serum-free medium for 24 hours. Images were taken at the same site after removal of the Culture-Insert and after 24 hours of incubation to assess cell migration.

### Transwell Assay

Transwell chambers with or without Matrigel (Corning Incorporated, NY, USA) were used to measure the migration and invasive capacity of CRC cells. Briefly, 5 × 104 CRC cells were resuspended in DMEM without FBS and seeded into the upper chamber and then DMEM with 10% FBS was added to the lower chamber. after 24 h of incubation, the cells in the upper chamber were removed and the migrating cells were stained with 0.1% crystal violet.

### Assessment of ferroptosis characteristics

2 × 10^5^ cells were seeded in six-well plates and treated for the indicated durations. To detect reactive oxygen species (ROS) and lipid ROS levels, cells were incubated in a PBS with DCFH-DA (Beyotime, China) and C11-BODIPY 581/591 (ABclonal, China). DCFH-DA (10μM) was used to detect ROS, with cells incubated in the dark for 30 min. C11-BODIPY 581/591 was used to detect lipid ROS, with cells incubated with 5 μM C11-BODIPY 581/591 for 30 min, after incubation, cells were digested into a single-cell suspension, and analyzed by flow cytometry (Beckman,USA). FerroOrange was used to detect intracellular Fe2⁺, following pretreatment with 1μM FerroOrange for 30 min, images were acquired under a confocal microscope (Carl Zeiss).

### Chromatin immunoprecipitation (ChIP)-qPCR

The ChIP experiment was conducted using a ChIP kit (Abcam, Shanghai, China) following the manufacturer’s instructions. RT-qPCR was then used to evaluate the ChIP signal, TableS1 presents the specific primers used in the ChIP-qPCR analysis.

### Dual-Luciferase Reporter assay

Cells were seeded into six-well plates and then transfected with the corresponding luciferase plasmid for 48 hours. The Dual-Luciferase Reporter Assay System (#11402ES, Yeasen) was used to perform the assay. The firefly luciferase activities were normalized to Renilla luminescence in each cell well.

### Immunohistochemistry (IHC)

The human CRC samples were collected from Zhejiang University Cancer Institute (ZUCI). All the patients were informed the aim for collecting tissue samples and consented the collection. This experiment was approved by the Ethics Committee of the Second Affiliated Hospital of Zhejiang University School of Medicine. IHC were employed to assess the levels of SPOP (#DF12106, Affinit,1:400) and β-catenin (#ab32572, Abcam, 1:600) in CRC tissue. The H-scores were divided into Density scores were categorized as 0 (negative), 1 (weak), 2 (moderate), and 3 (strong): Density scores were 0-5% = 1, 6-30% = 2, 31-70% = 3, and 71-100% = 4. The two scores were then multiplied to give a total IHC score (0 to 12). 0-6 was classified as low expression, and scores of 6 or more were classified as high expression. All cases were scored by two independent pathologists.

**Supplementary Table S1. Primers for qPCR and ChIP-qPCR**

| **qPCR Primers** | | |
| --- | --- | --- |
| Gene | | Sequence (5’to 3’) |
| SPOP | F | GGAAGGCTCCAAACCTCGACAA |
|  | R | AGCGTTCTCCACGGACAGGTTA |
| GAPDH | F | TTGCCCTCAACGACCCTTT |
|  | R | TCCTCTTGTGCTCTTGCTGG |
| CTNNB1 | F | AAAGCGGCTGTTAGTCACTGG |
|  | R | CGAGTCATTGCATACTGTCCAT |
| SLC7A11 | F | TCTCCAAAGGAGGTTACCTGC |
|  | R | AGACTCCCCTCAGTAAAGTGAC |
| CHIP-SLC7A11 promote | F | AGCTGAGTAATGCTGGAGGC |
|  | R | CTCAGCTTCCTCATGGGCTT |

**Supplementary Table S2. The shRNA sequences in the study.**

| For shRNA construction |  |
| --- | --- |
| shSPOP-1 | 5'- CAAACGCCTGAAGCAATCCTA-3' |
| shSPOP-2 | 5'-CACAGATCAAGGTAGTGAAAT-3' |
| sh-CTNNB1 | 5’-TCTAACCTCACTTGCAATAAT-3’ |
| shOGT-1 | 5’-GCCCTAAGTTTGAGTCCAAAT-3’ |
| ShOGT-2 | 5’-GCTGAGCAGTATTCCGAGAAA-3’ |

## Supplementary Figures

**
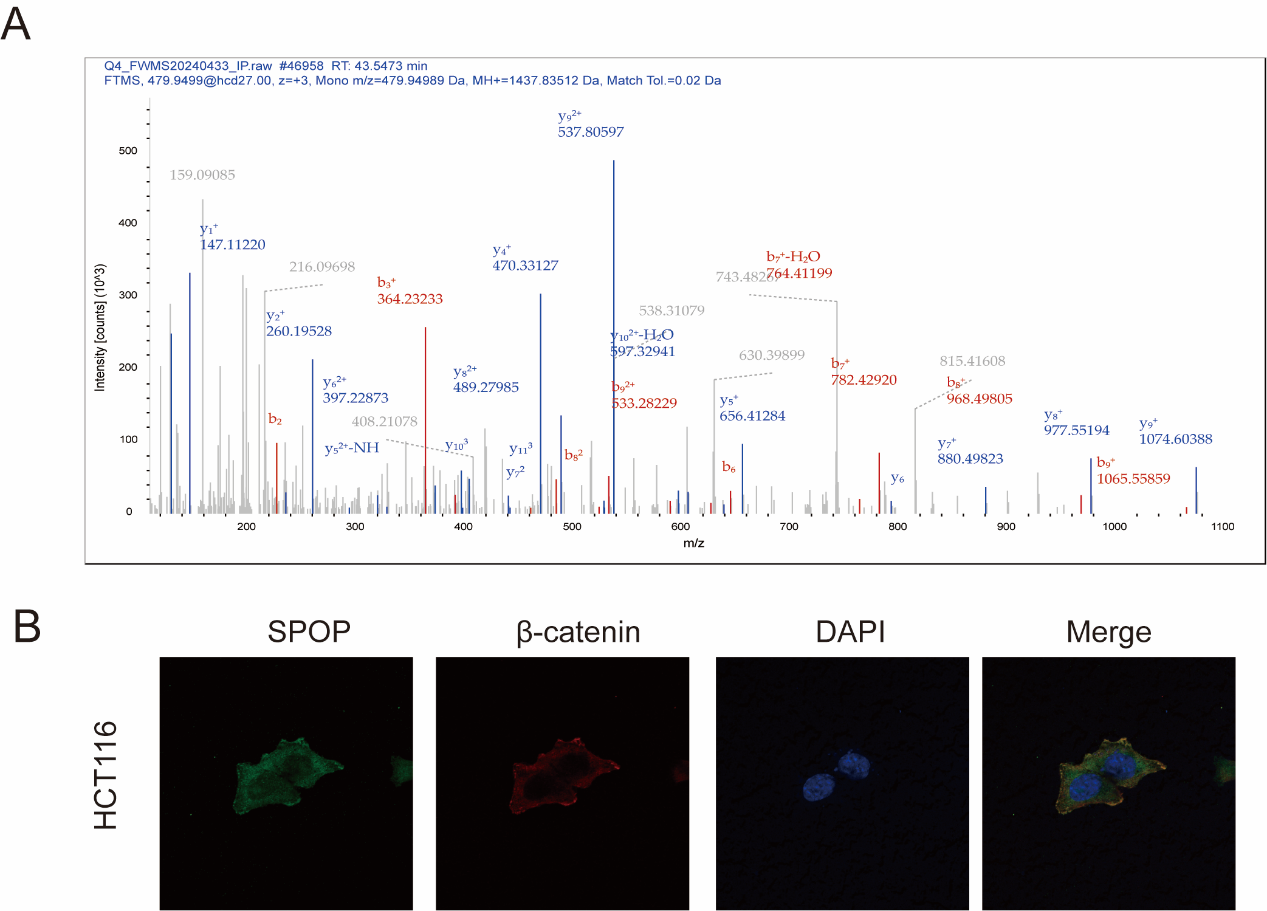
**

**Figure S1.** (A) Secondary mass spectrometry diagrams of β-catenin.(B) IF detection of co-localization of SPOP and β-catenin in HCT116 cells


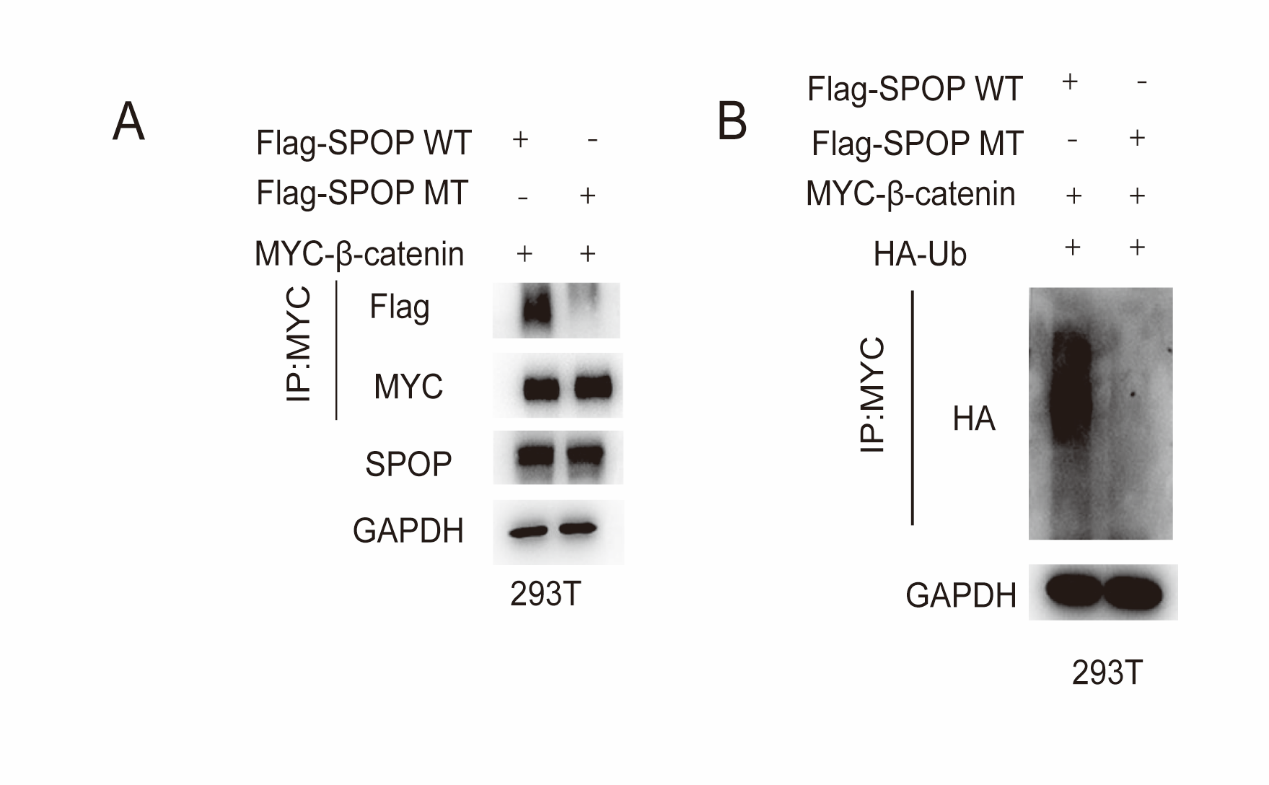


**Figure S2.** (A) 293T cells were transfected with Flag-SPOP-WT, Flag-SPOP-MT and MYC-β-catenin, and cell lysates were subjected to IP analysis using anti-MYC antibody. (B) 293T cells were transfected with Flag-SPOP-WT, HA-Ub and MYC-β-catenin, and cell lysates were analyzed by IP using anti-MYC antibody.


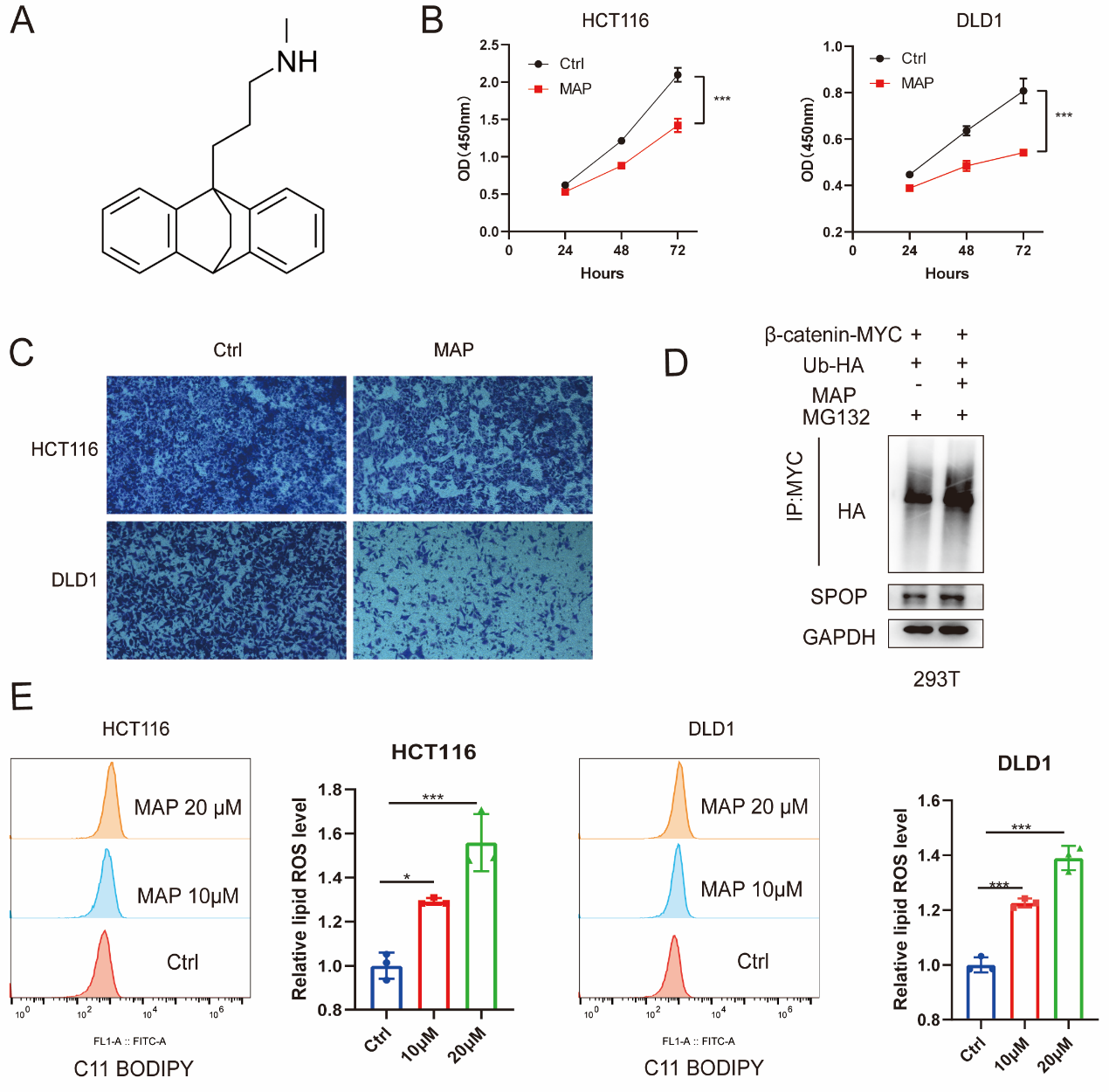


**Figure S3.**

(A) Maprotiline Chemical Structure.(B) CCK-8 assay of HCT116 and DLD1.

(C) Transwell assay of HCT116 and DLD1. (D) 293T cells were transfected with HA-Ub and MYC-β-catenin, treated by MAP for 24 h. Cell lysates were analyzed by IP using anti-MYC antibody. (E) C11-BODIPY probe staining and lipid peroxidation levels were assessed by flow cytometry. Data are presented as mean ± SD. Statistical significance was determined by Student's t-test and ANOVA test. *p < 0.05, **p < 0.01, ***p < 0.001.

**
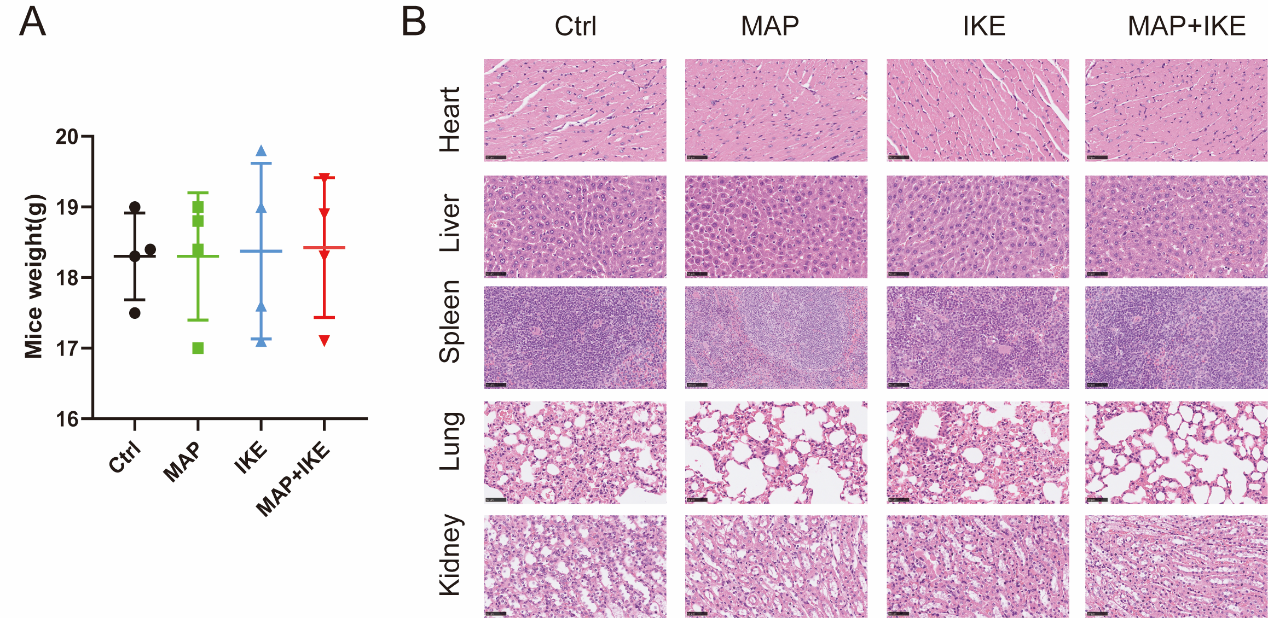
**

**Figure S4.** (A) Body weight measurements of tumor-bearing mice. (B) Representative H&E staining of heart, kidney and lung tissues form all treatment groups，scale bar 50 μm. Data are presented as mean ± SD.
