# Supplementary material for: O-GlcNAcylation of SPOP regulates colorectal cancer progression and ferroptosis by mediating β-catenin degradation
Source: Cell Death Discov. 2025 Nov 10;11:526. doi: 10.1038/s41420-025-02832-y (PMC12603323; doi:10.1038/s41420-025-02832-y)
Supplement: Supplementary file 3 — Original western blots [file 41420_2025_2832_MOESM3_ESM.pdf]

Fig.2A

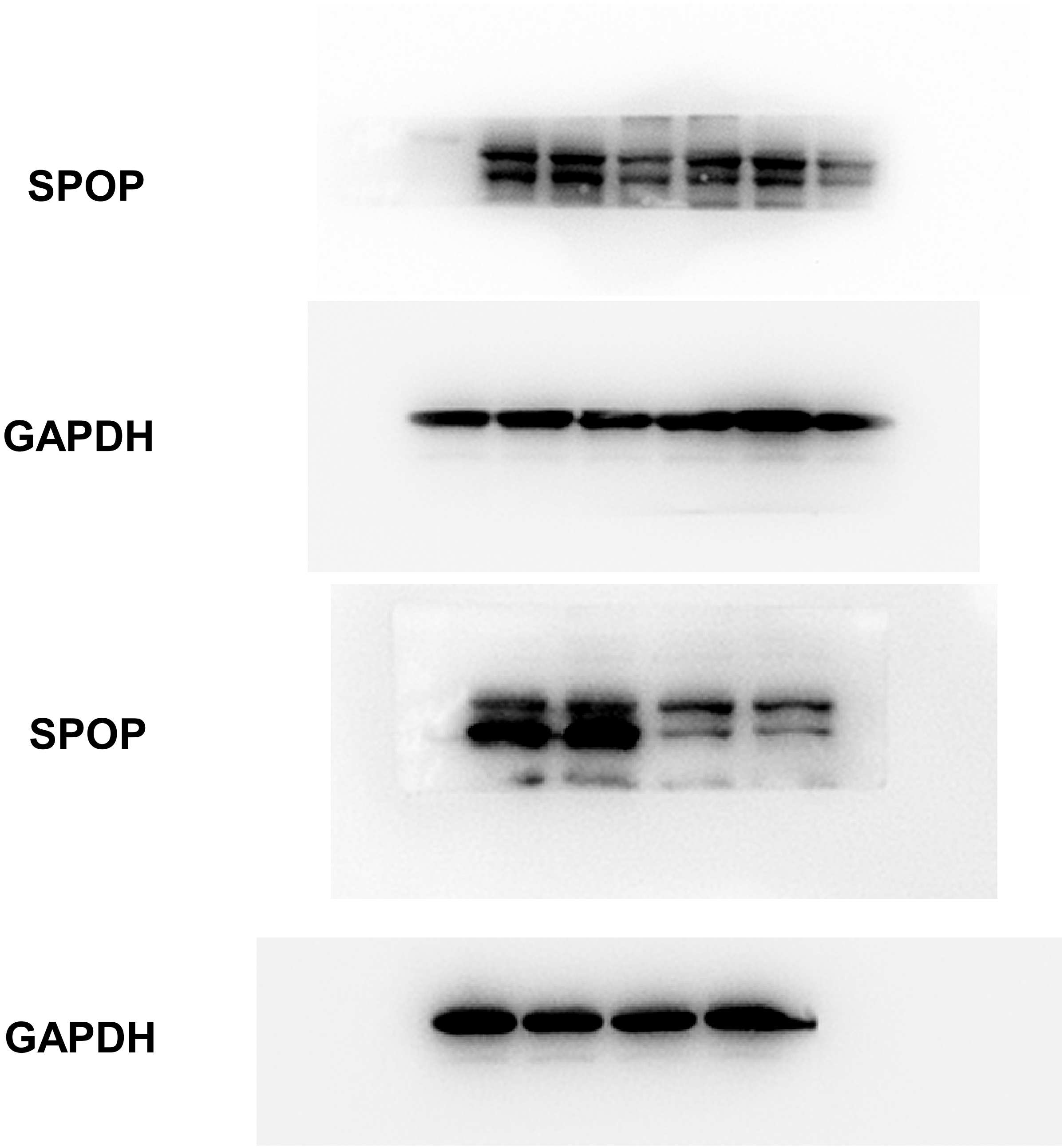

Fig.3E

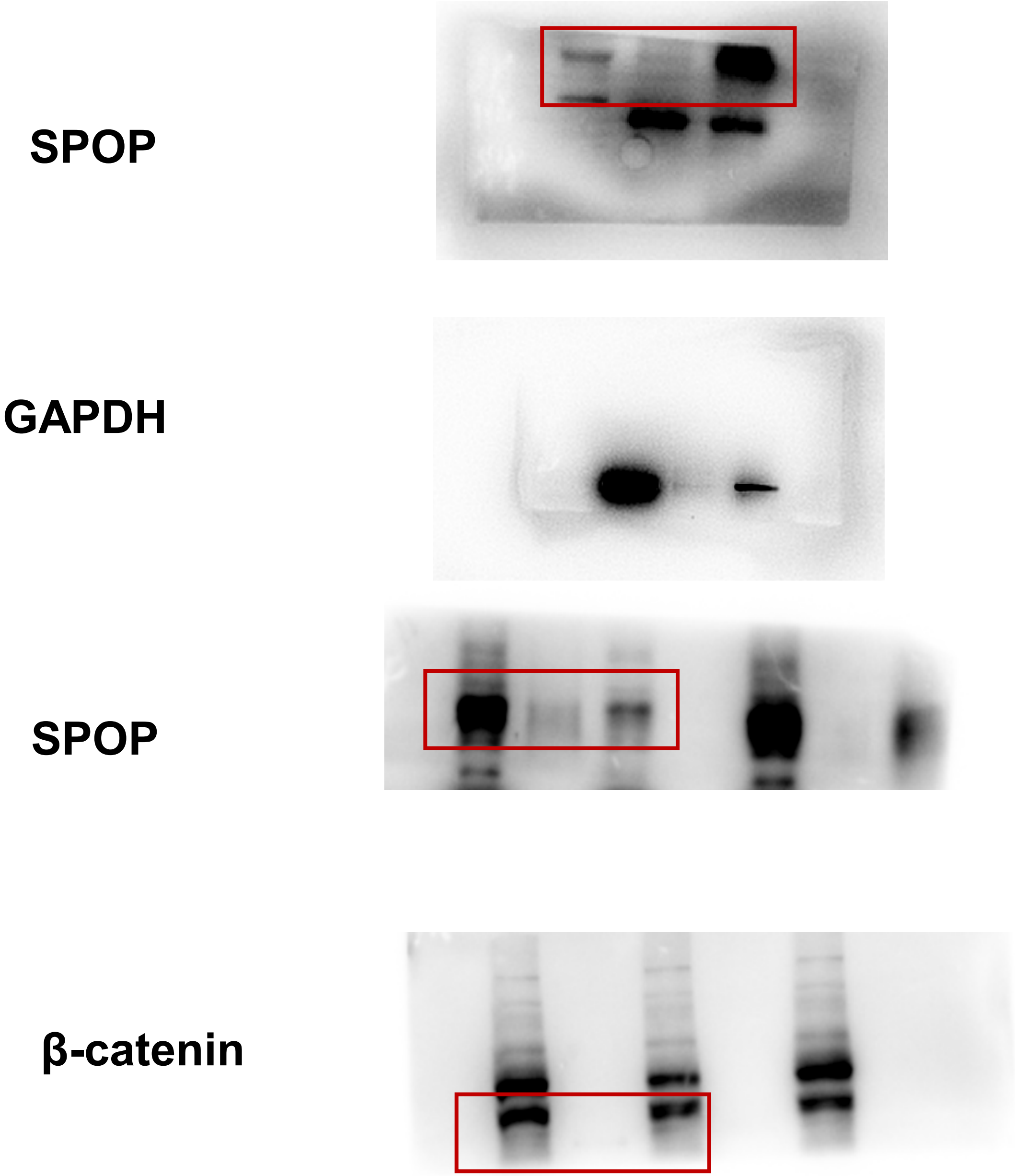

Fig.3F

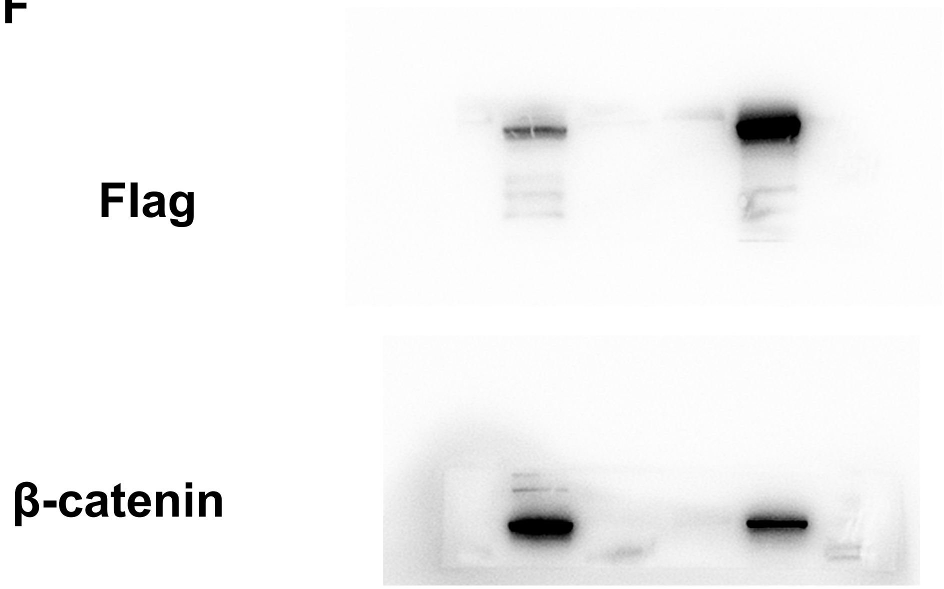

Fig.3G

Flag

MYC

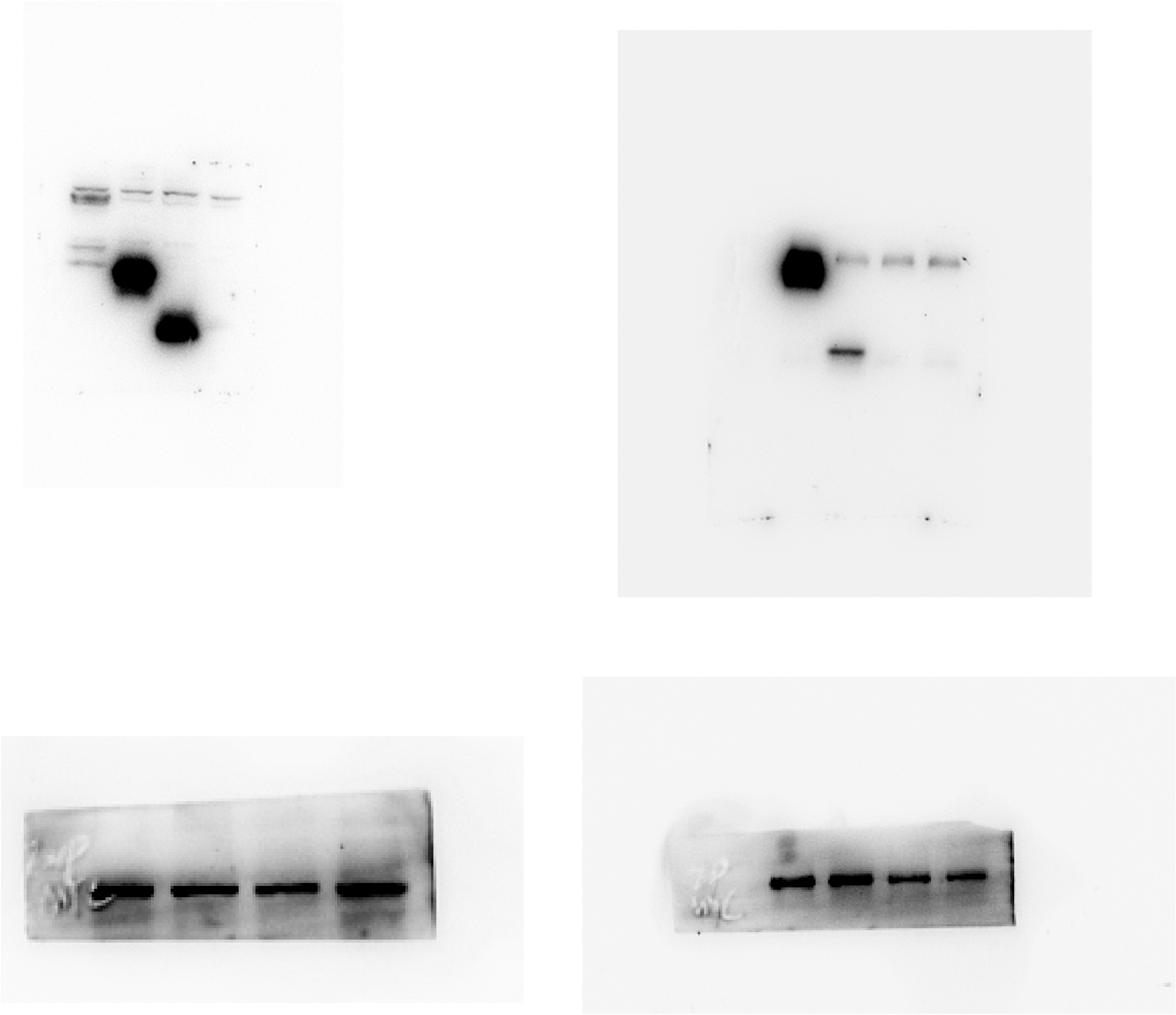

Fig.4B

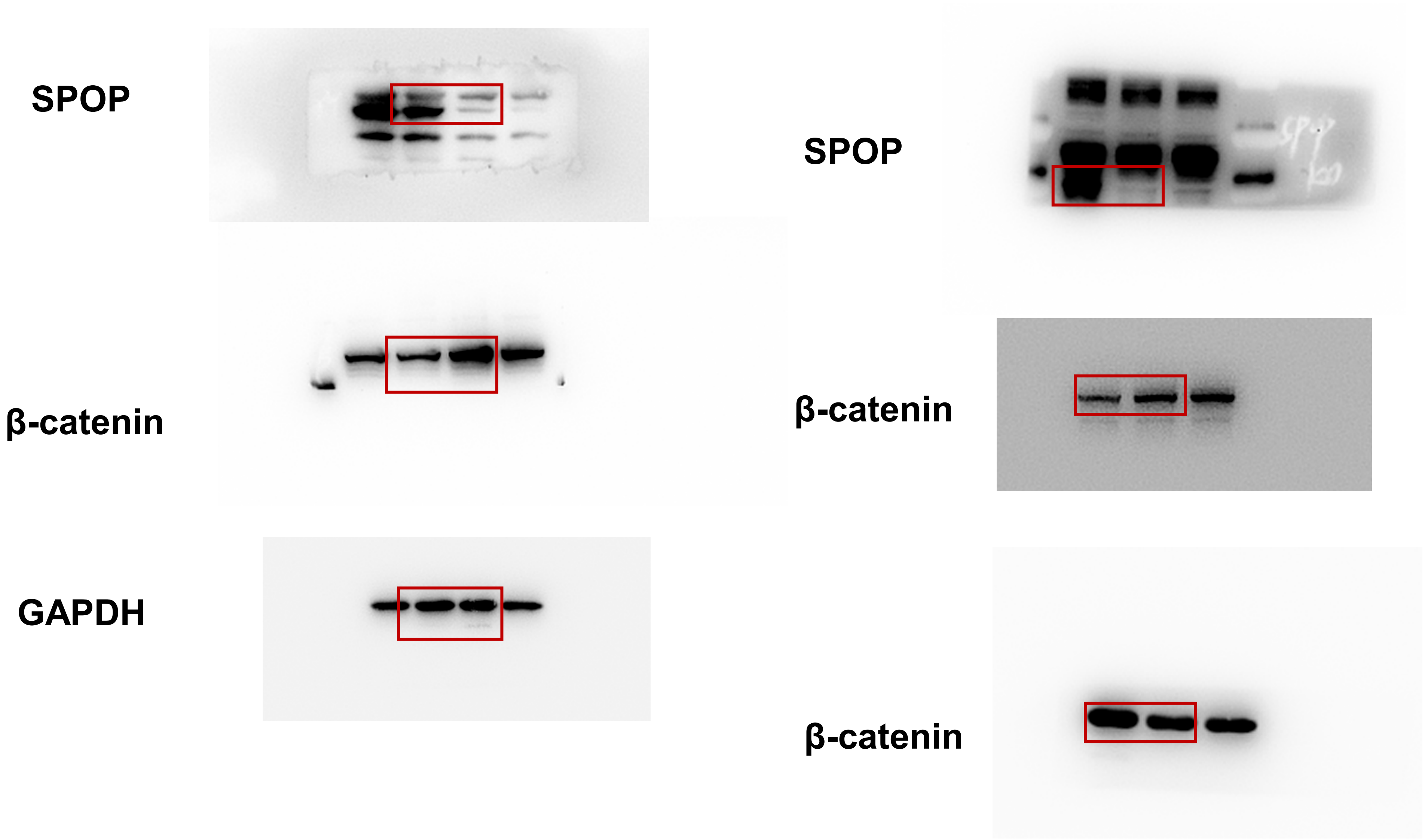

Fig.4B

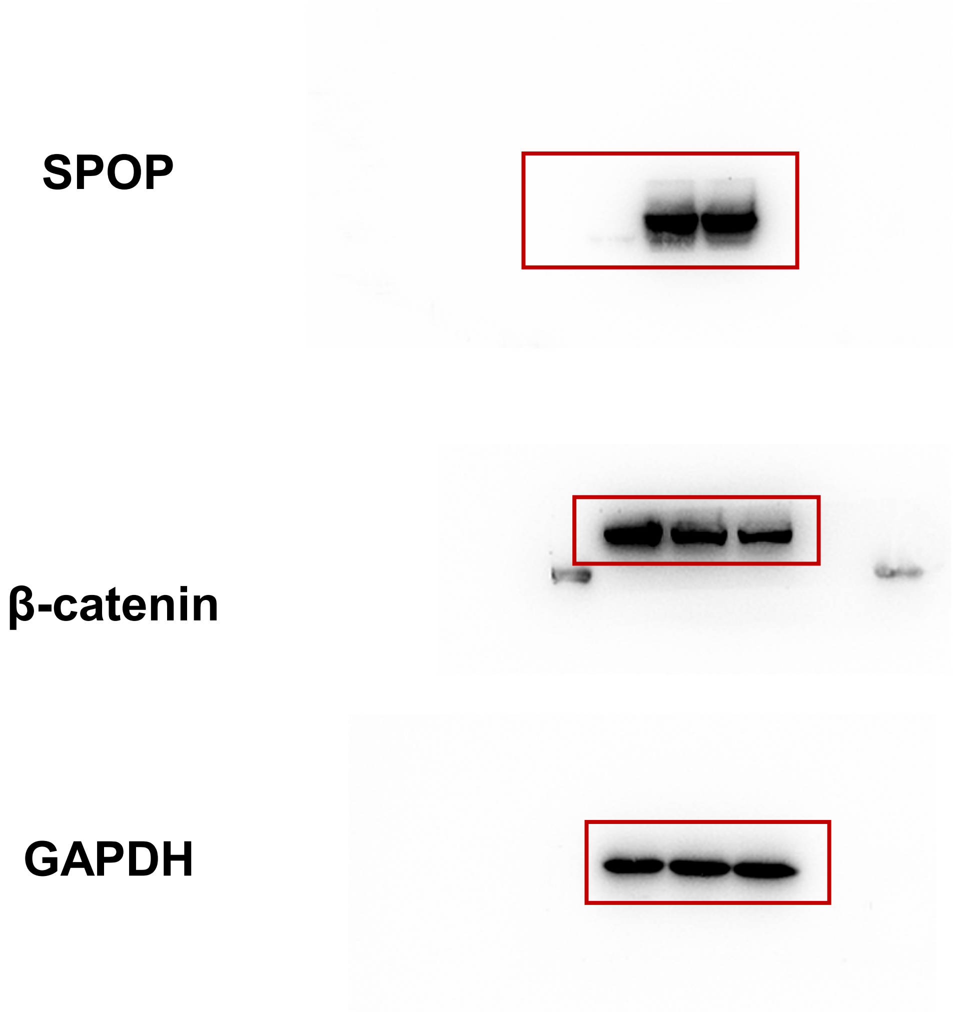

Fig.4C

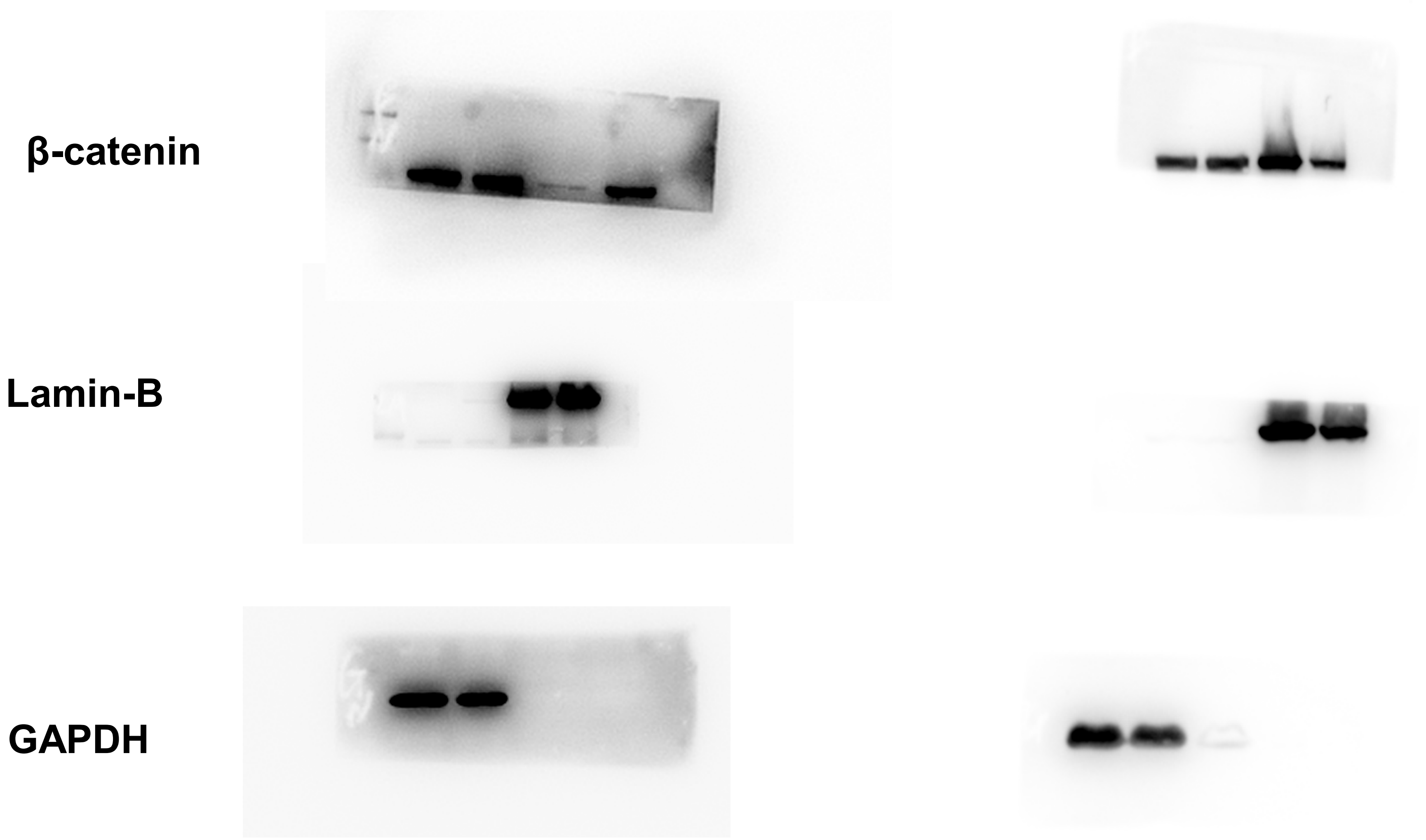

Fig.4E

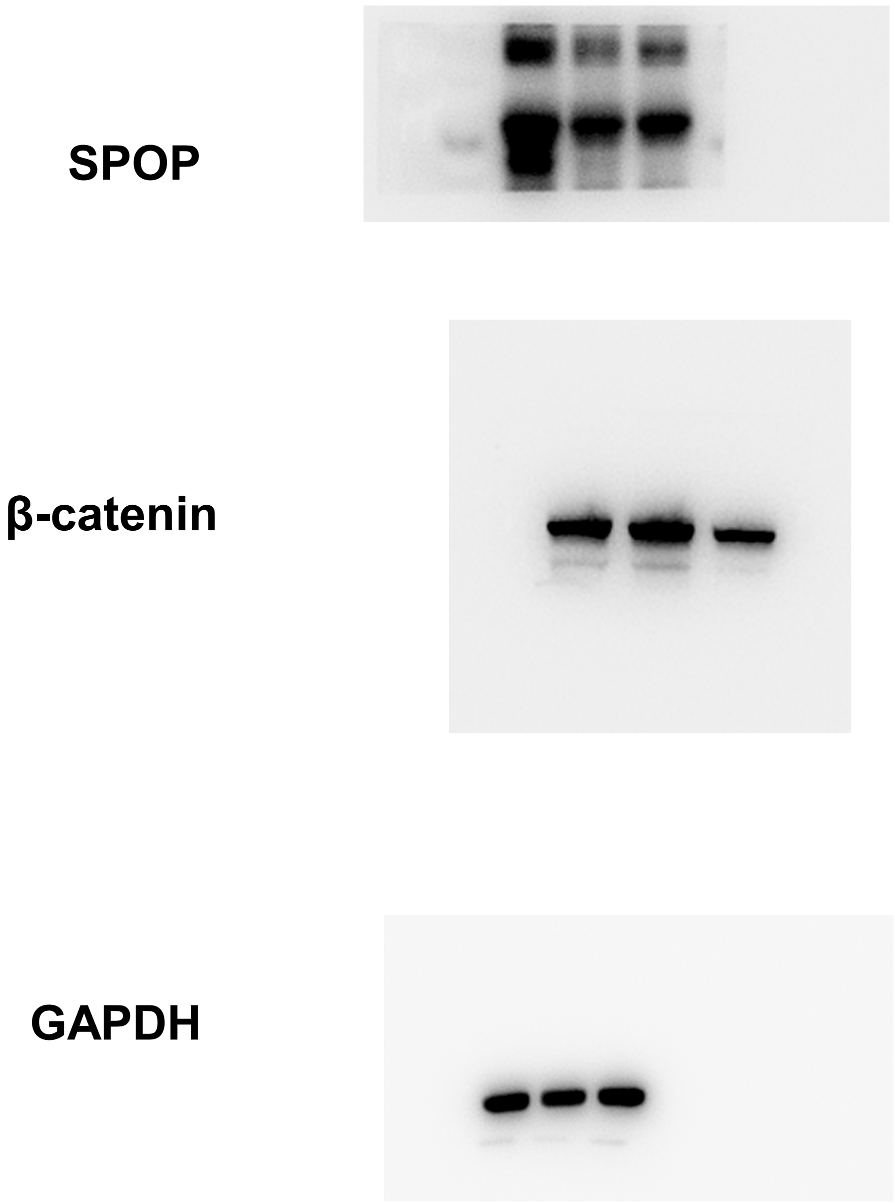

Fig.5A

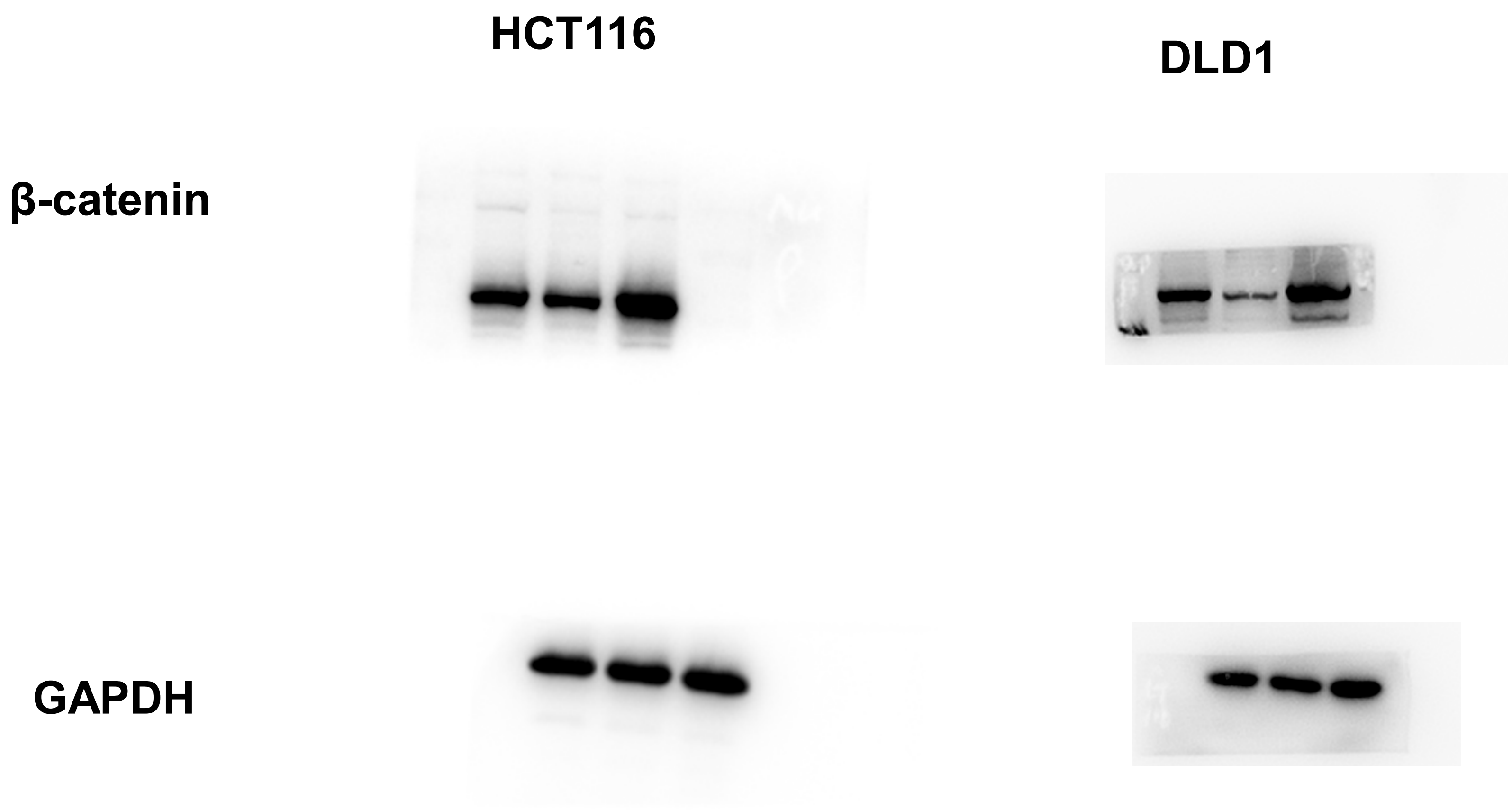

Fig.5B

β-catenin

GAPDH

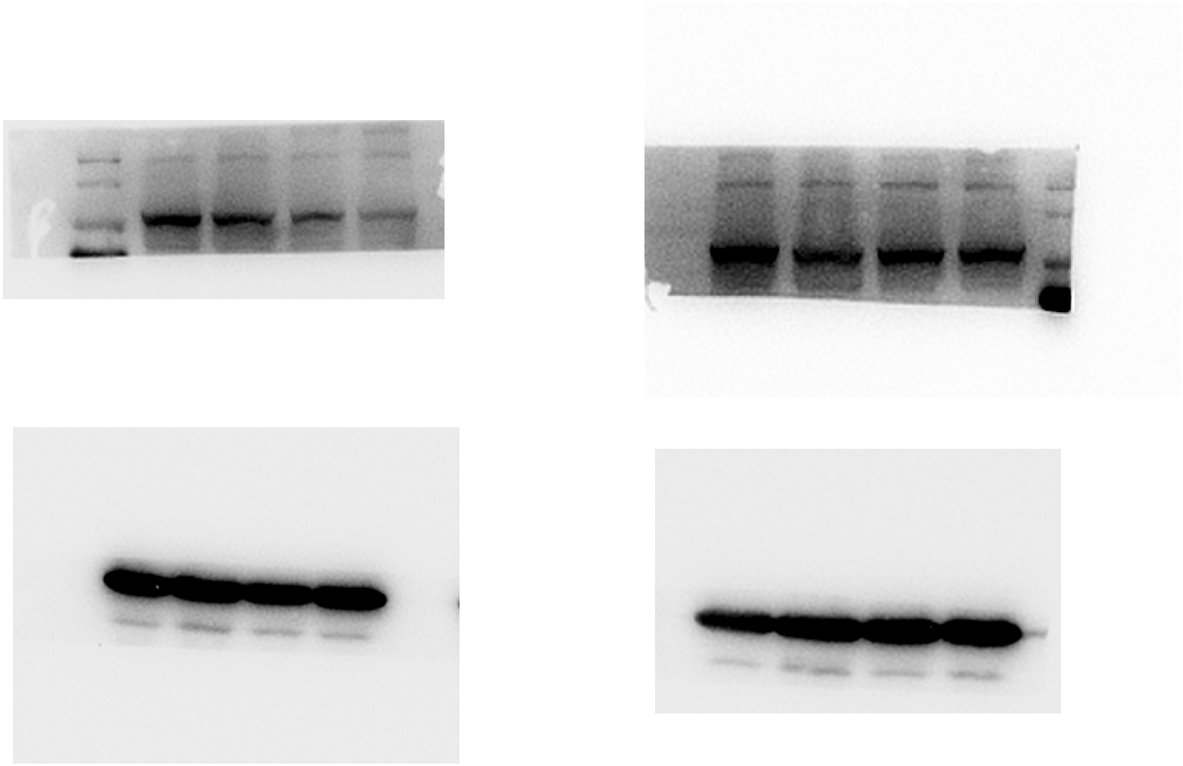

Fig.5C

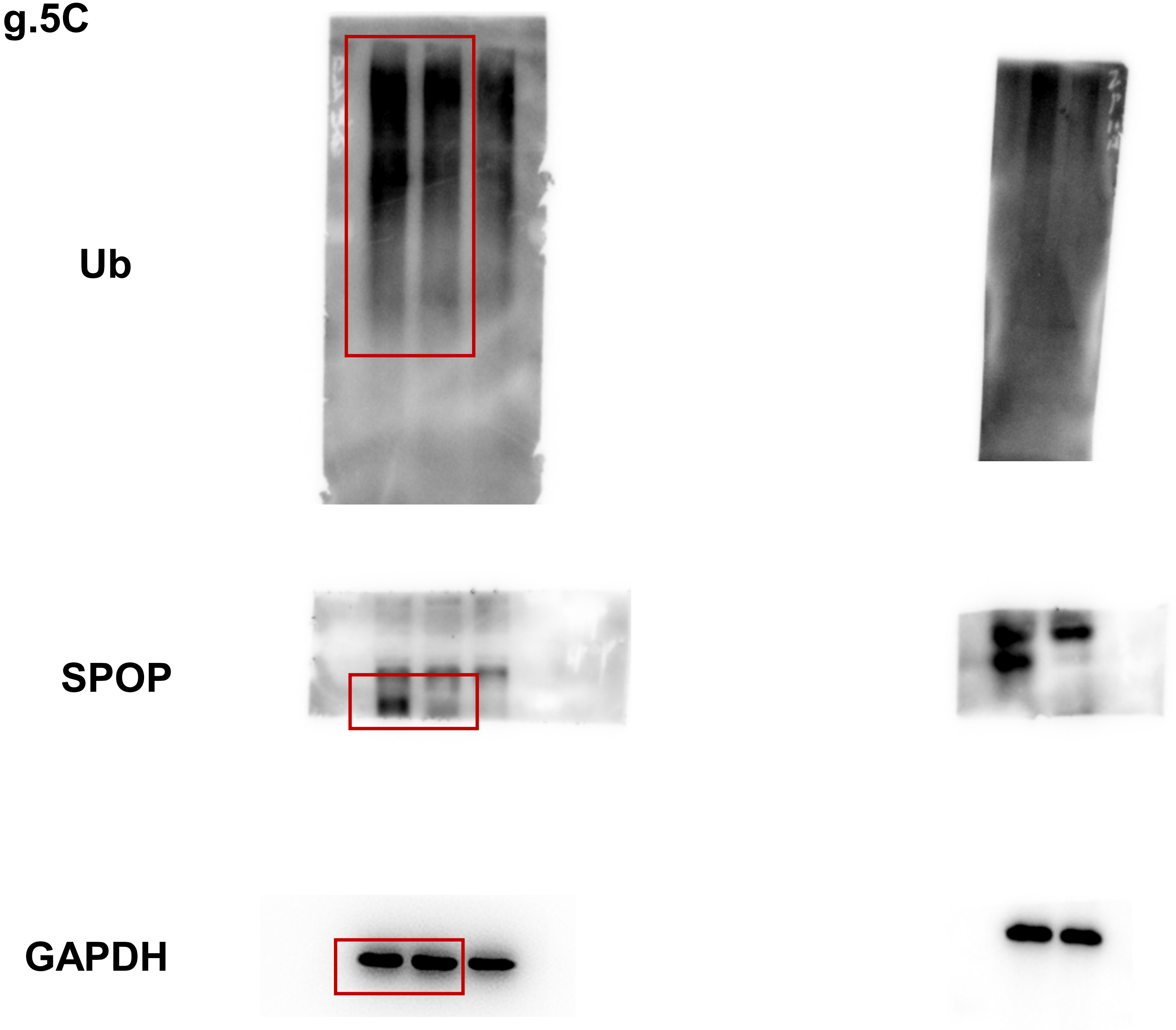

Fig.5D

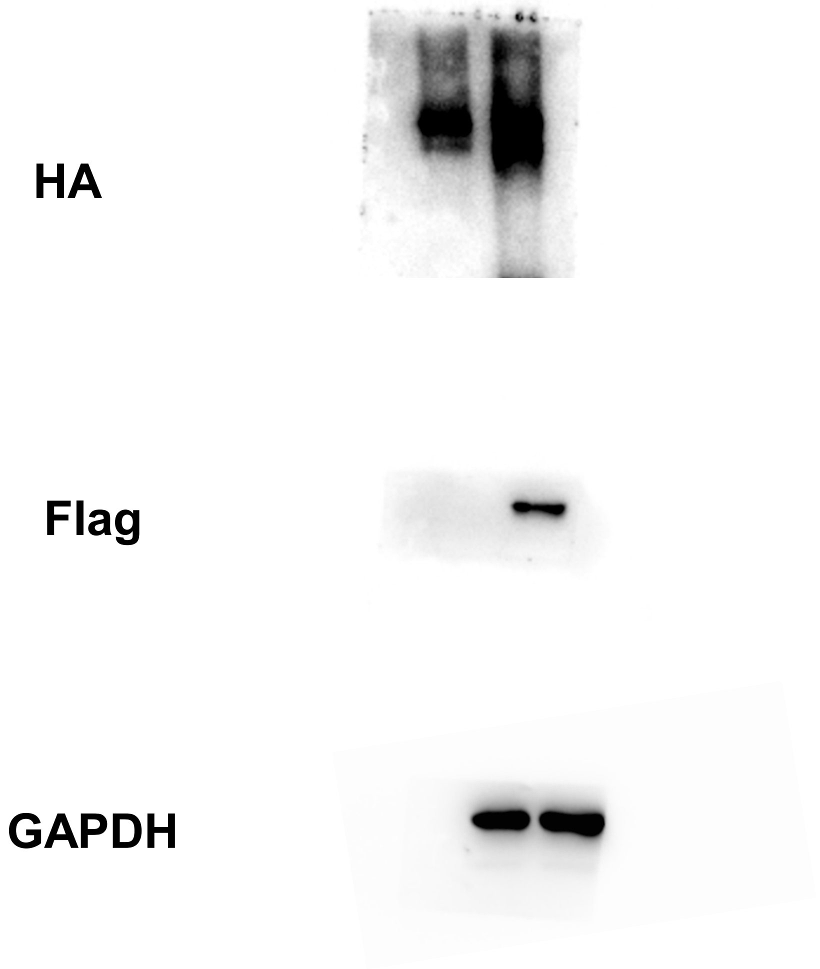

Fig.5F

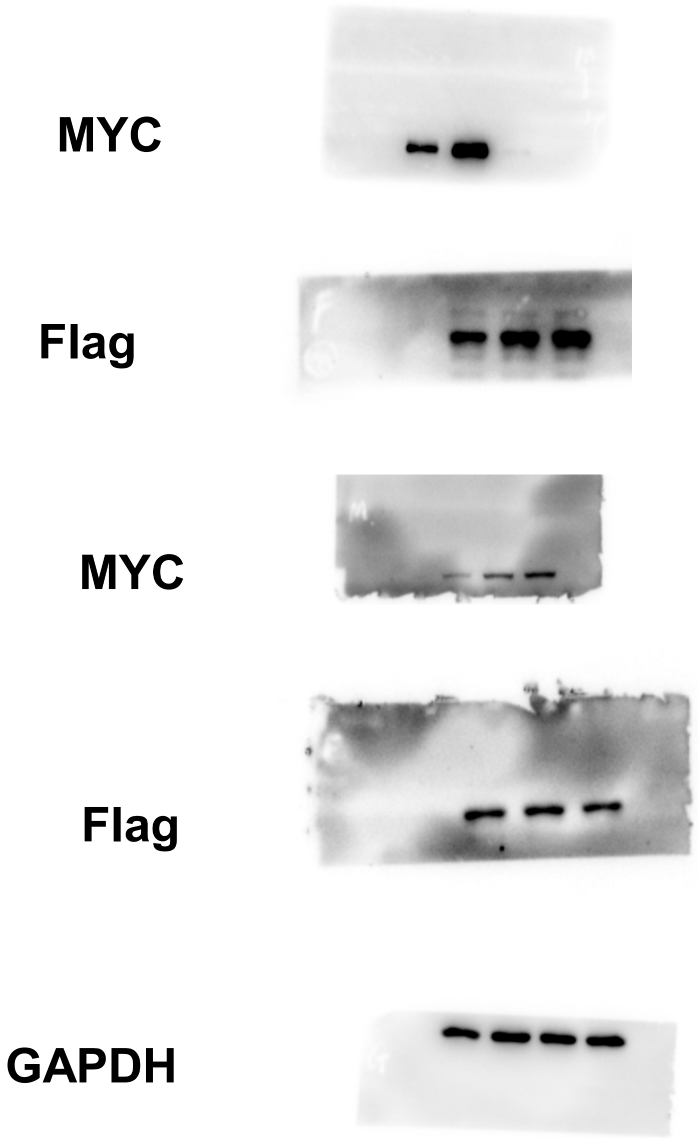

Fig.5G

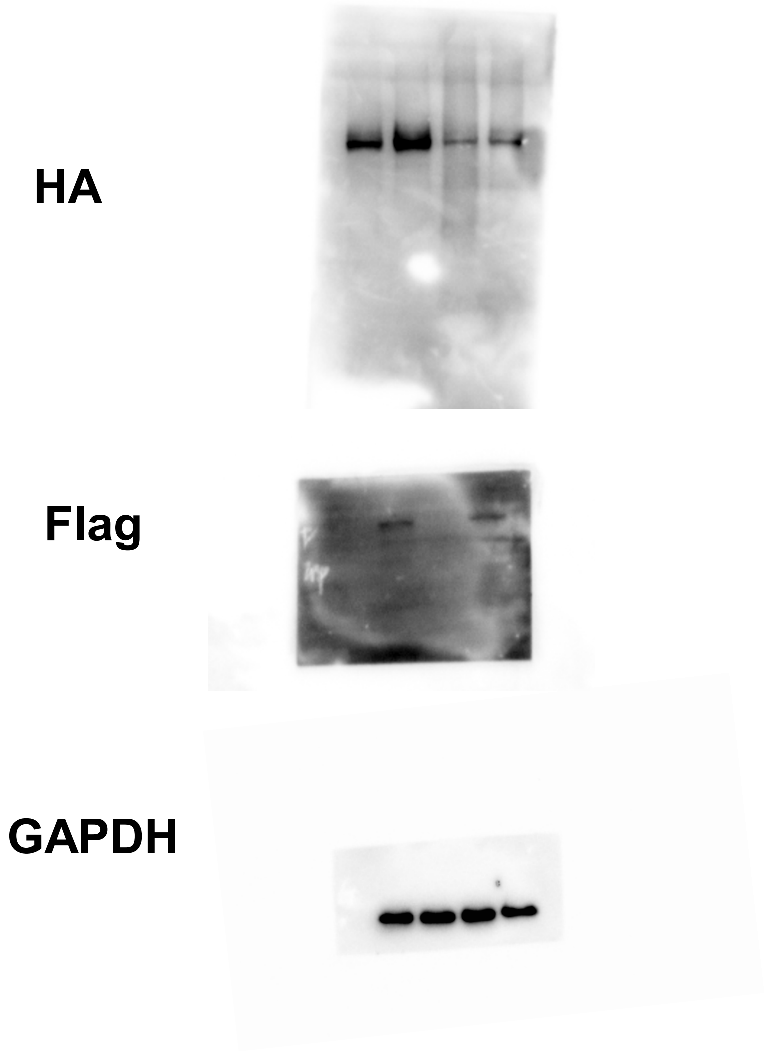

Fig.5H

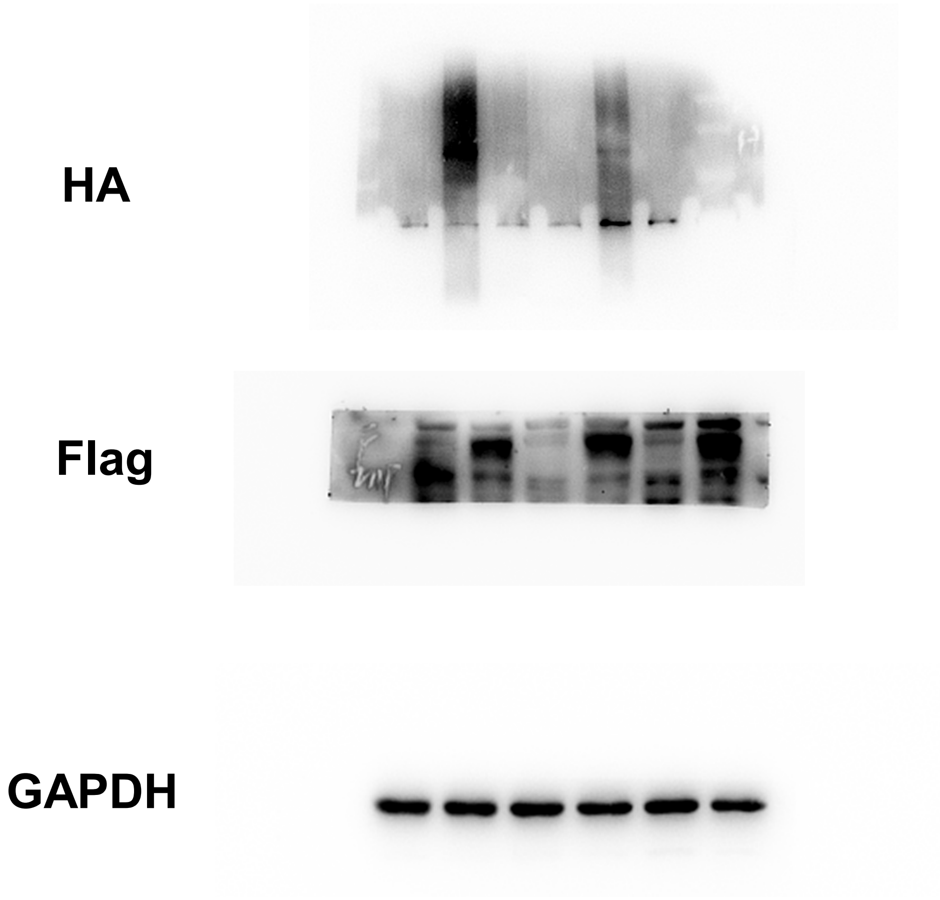

Fig.5I

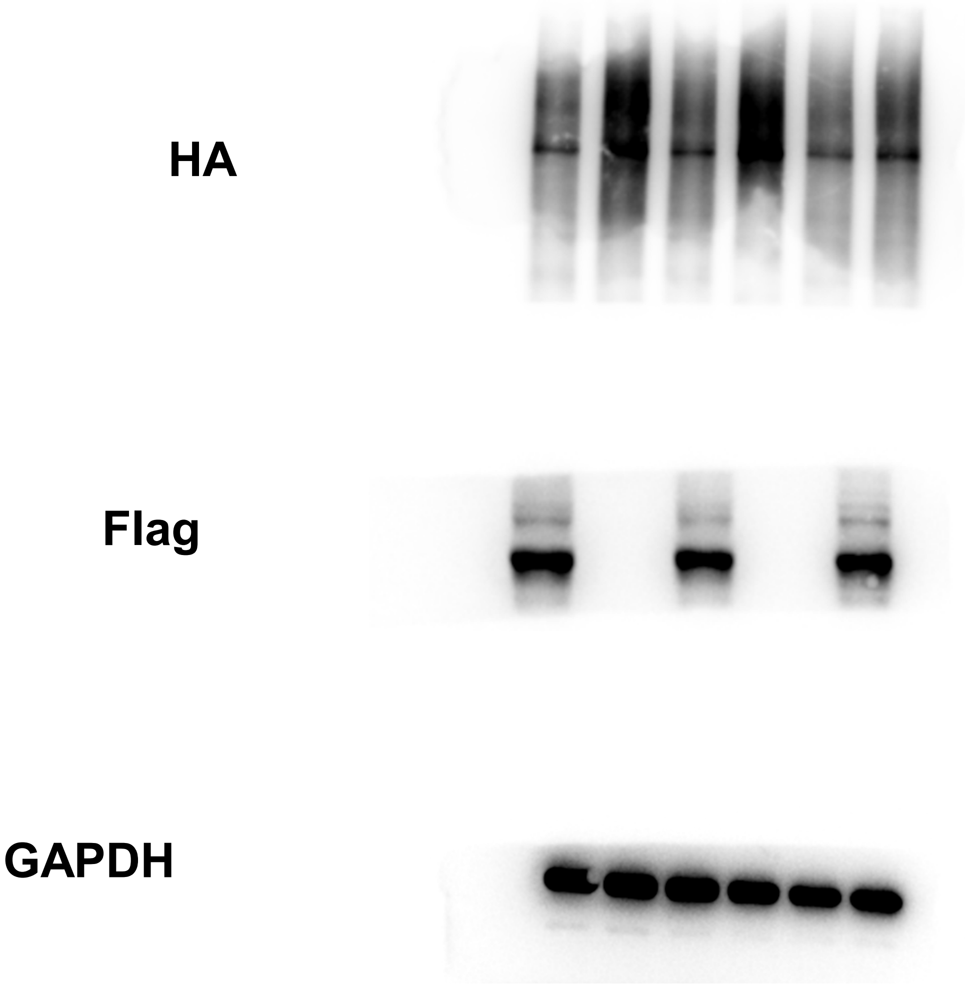

Fig.6A

OGT

SPOP

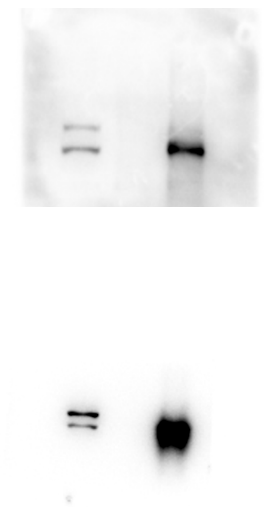

Fig.6B

OGT

O-glc

SPOP

GAPDH

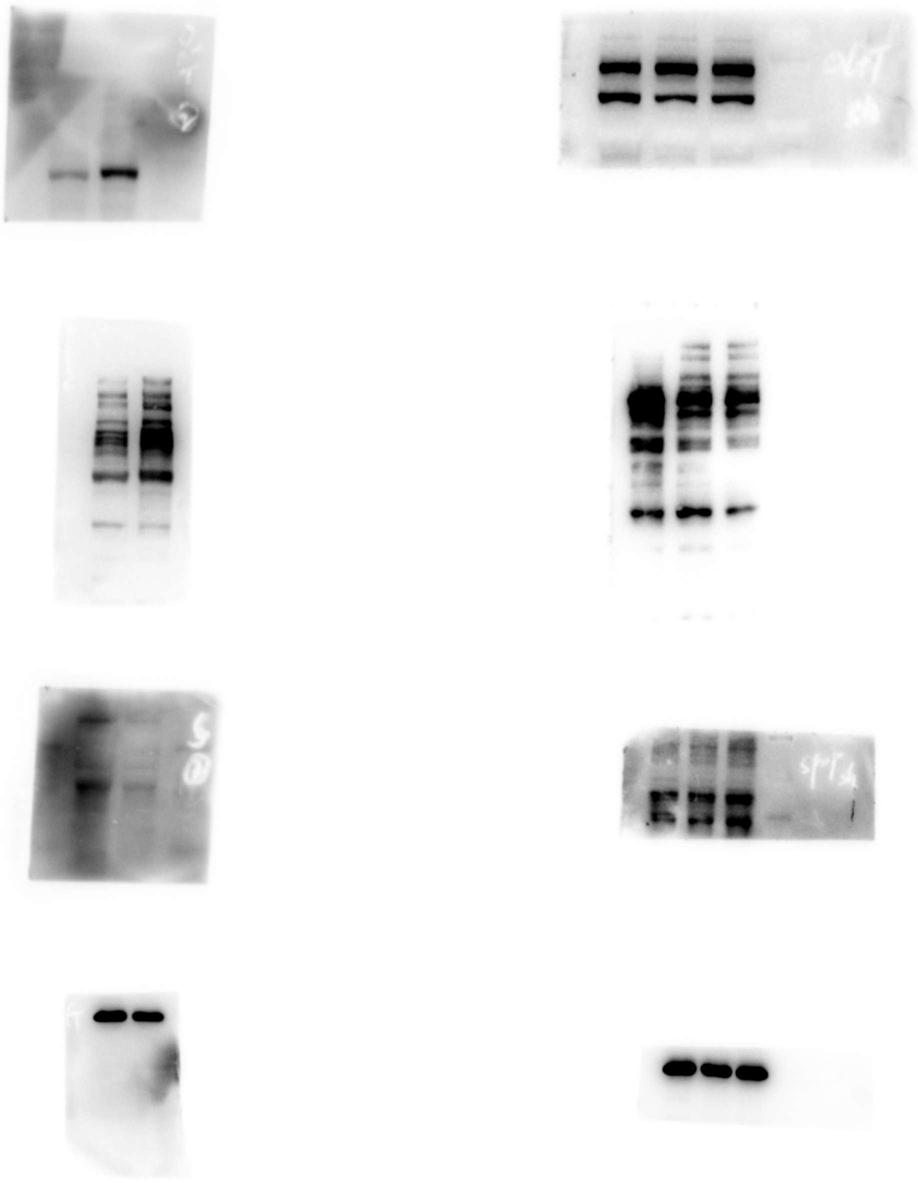

Fig.6D

O-glc

SPOP

GAPDH

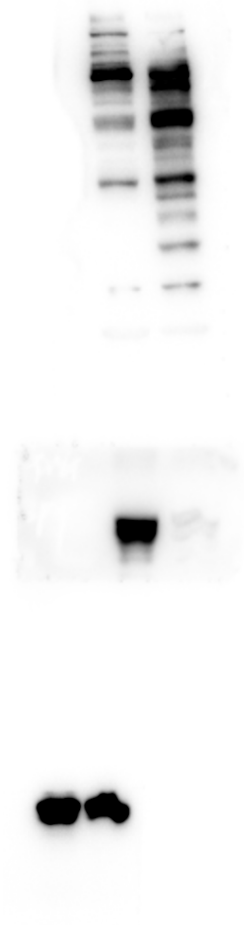

Fig.6E

OGT

SPOP

GAPDH

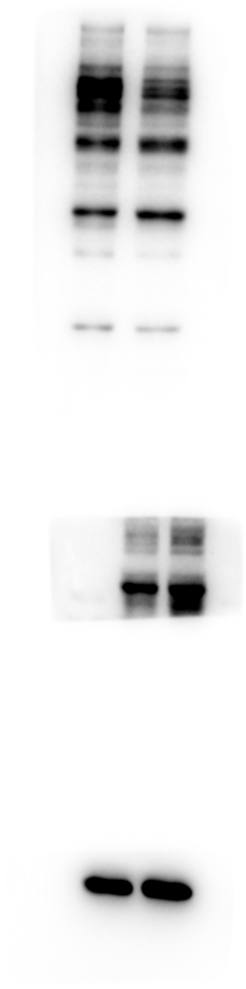

Fig.6F

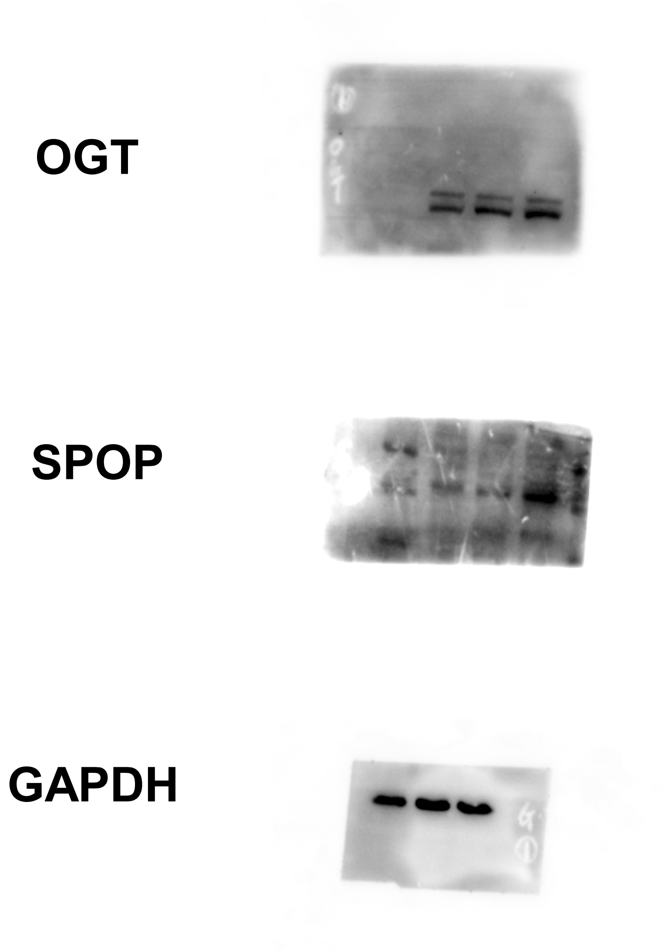

Fig.6G

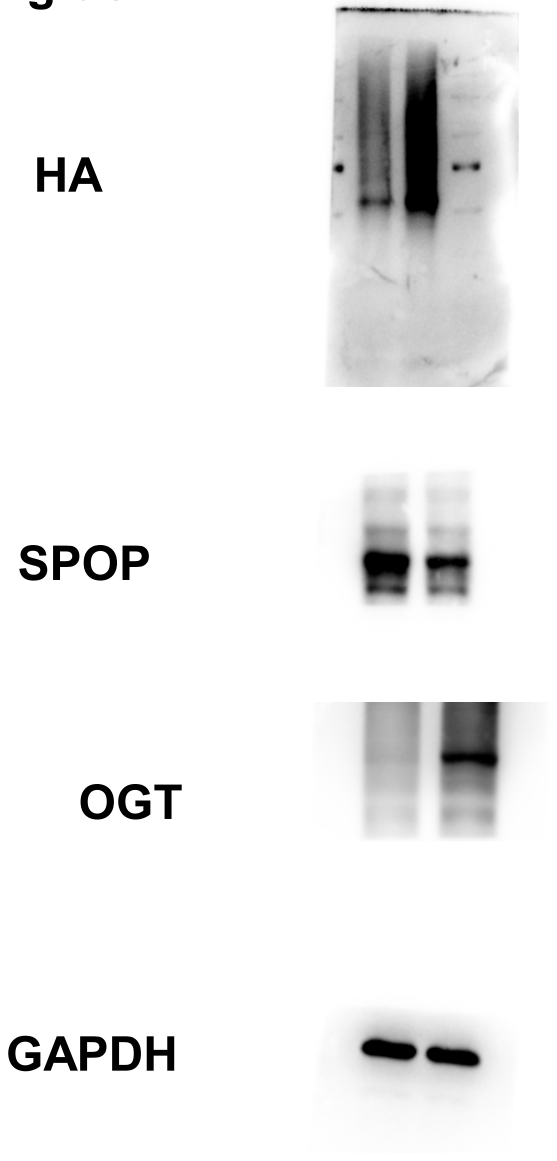

Fig.6I

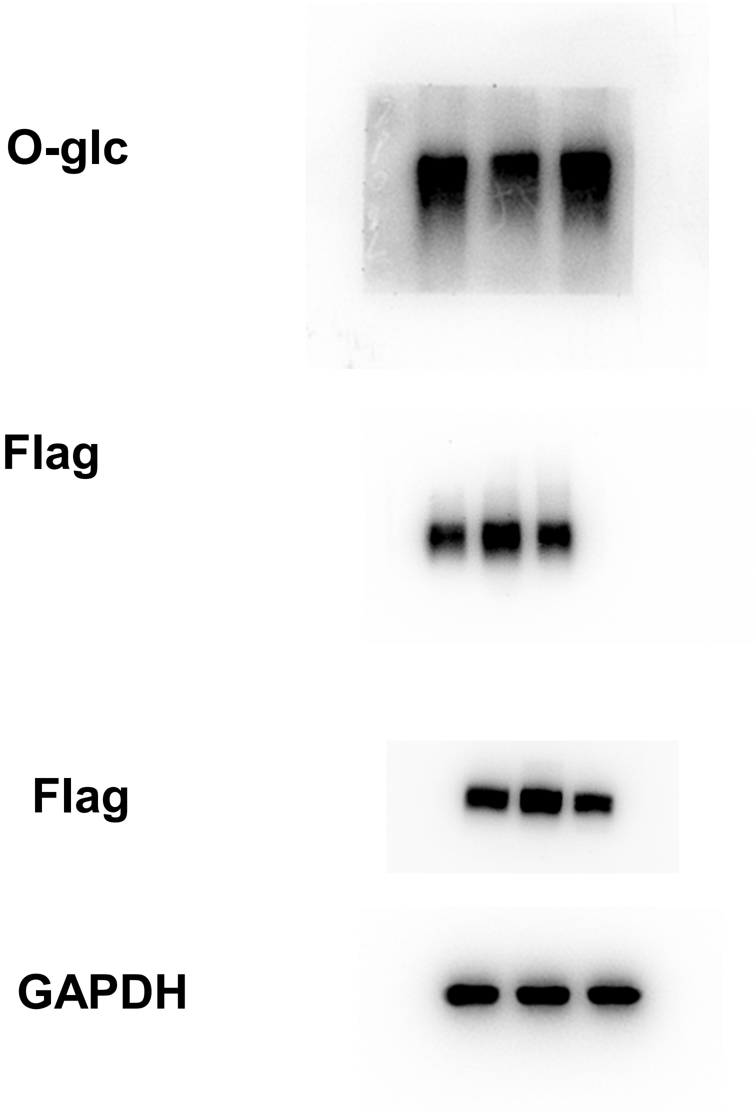

Fig.6J

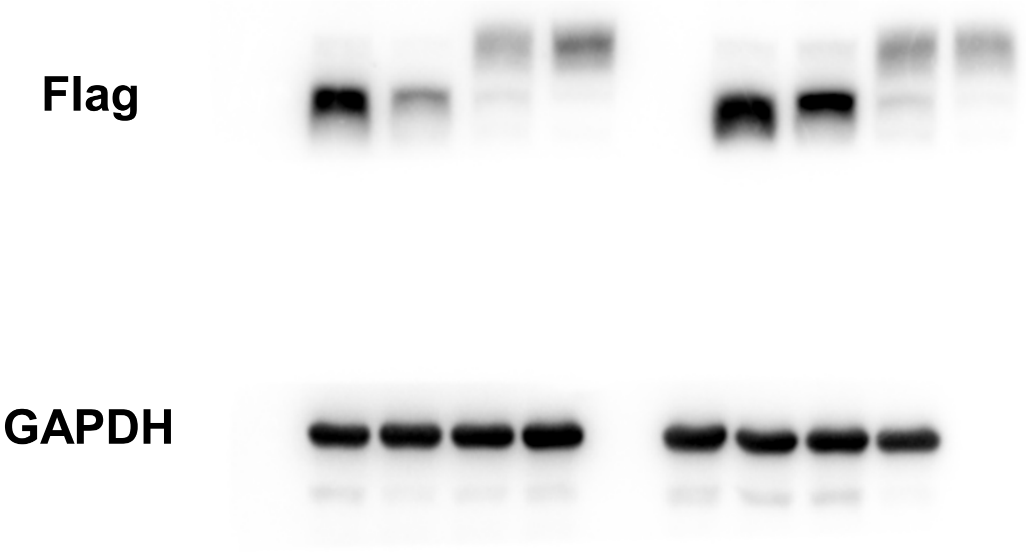

Fig.6K

HA

Flag

GAPDH

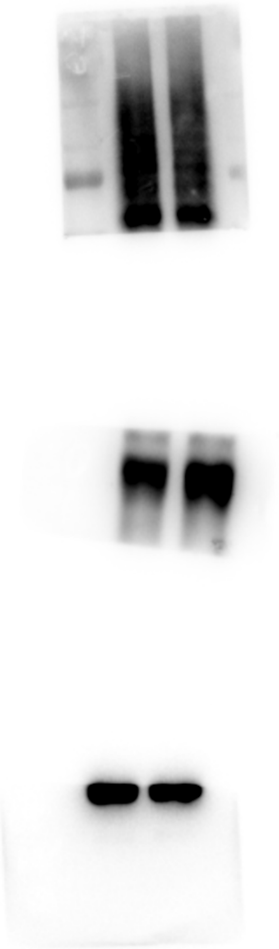

Fig.6L

β-catenin

GAPDH

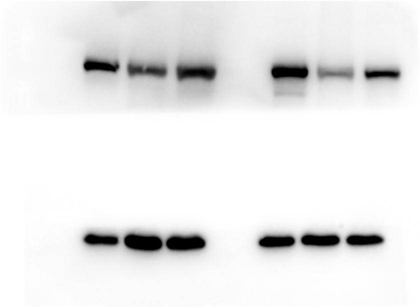

Fig.6M

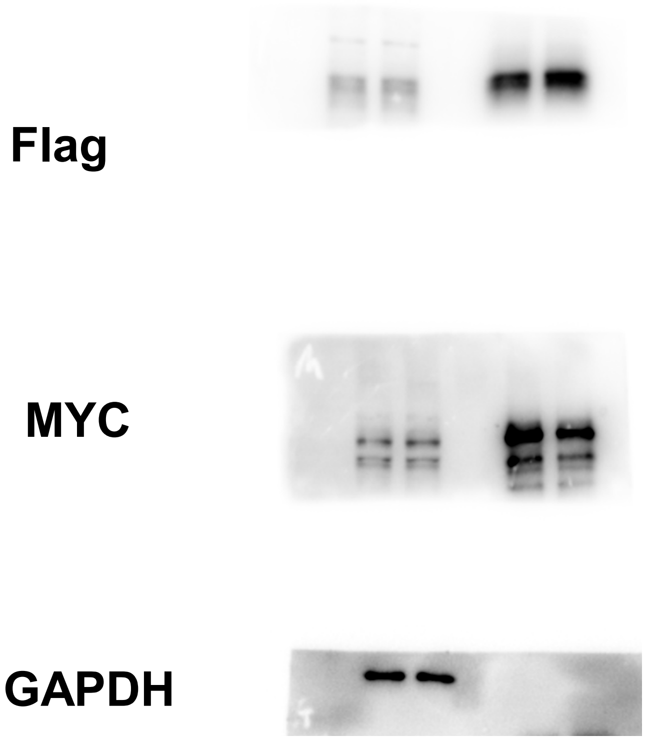

Fig.6N

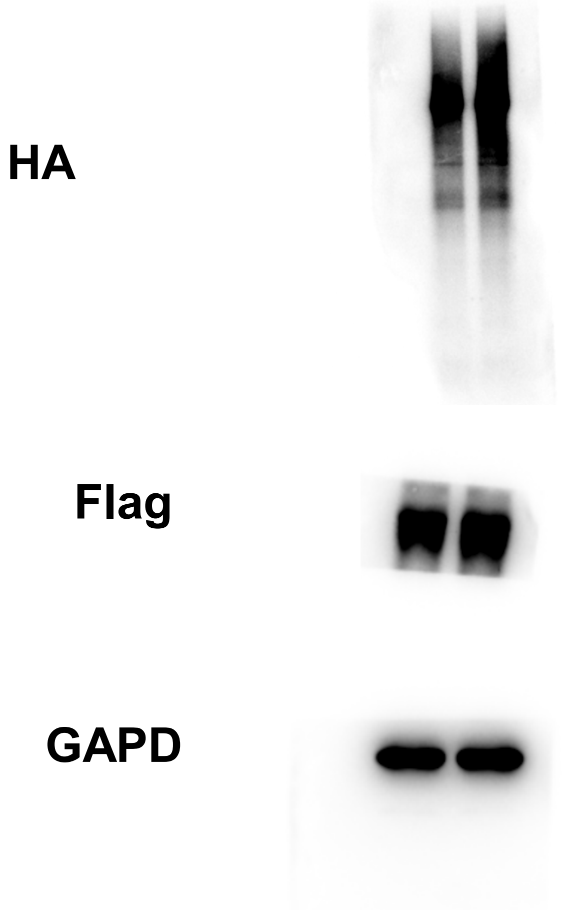

Fig.8K

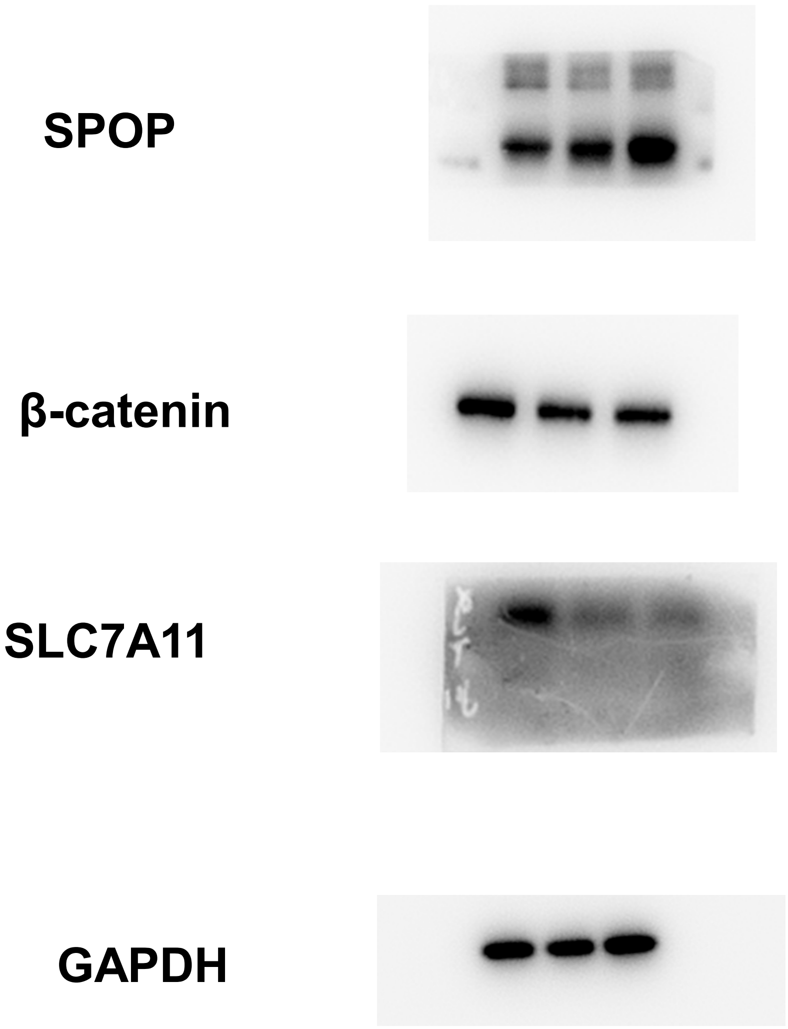

Fig.S2A

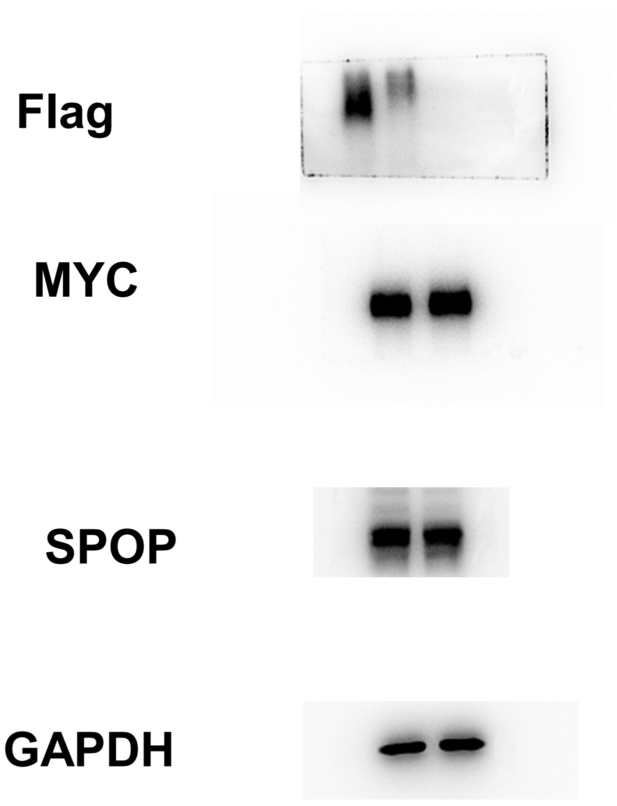

Fig.S2B

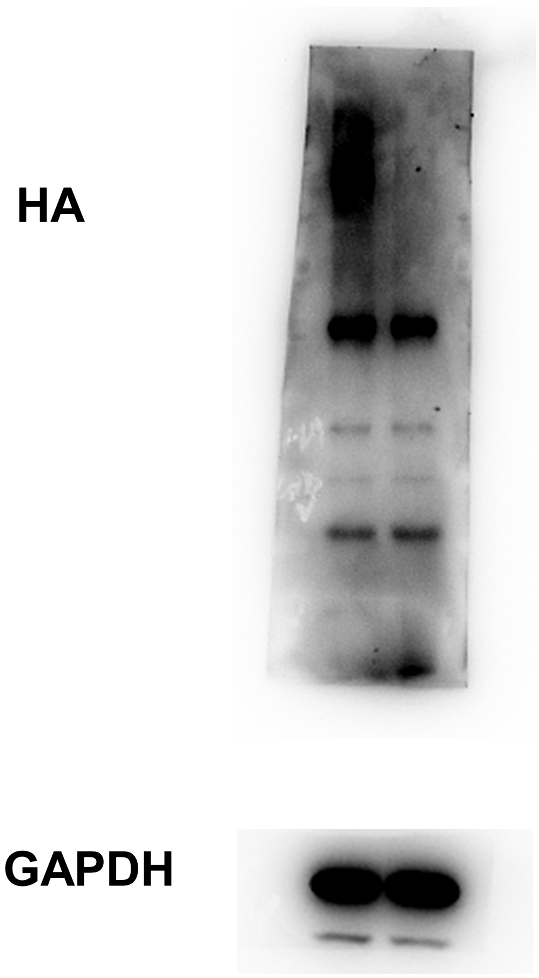

Fig.S3

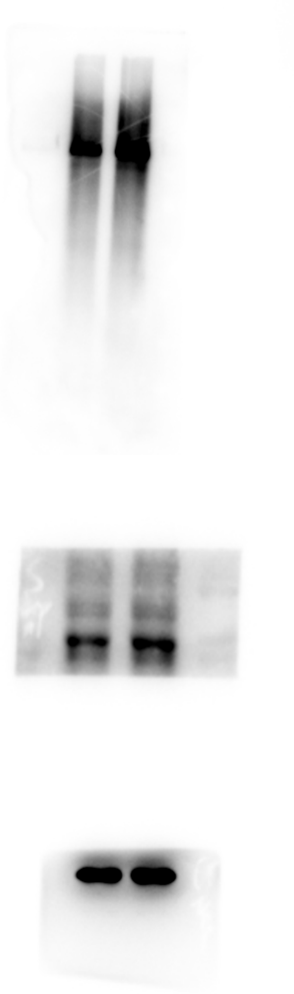

HA

SPOP

GAPDH
